# Molecular Characterization of Secreted Factors and Extracellular Vesicles-Embedded miRNAs from Bone Marrow-Derived Mesenchymal Stromal Cells in Presence of Synovial Fluid from Osteoarthritis Patients

**DOI:** 10.3390/biology11111632

**Published:** 2022-11-08

**Authors:** Enrico Ragni, Carlotta Perucca Orfei, Federico Valli, Luigi Zagra, Laura de Girolamo

**Affiliations:** 1 Laboratorio di Biotecnologie applicate all’Ortopedia, IRCCS Istituto Ortopedico Galeazzi, Via R. Galeazzi 4, I-20161 Milan, Italy; 2 Chirurgia Articolare Sostitutiva e Chirurgia Ortopedica (CASCO), IRCCS Istituto Ortopedico Galeazzi, Via R. Galeazzi 4, I-20161 Milan, Italy; 3 Hip Department, IRCCS Istituto Ortopedico Galeazzi, Via R. Galeazzi 4, I-20161 Milan, Italy

**Keywords:** osteoarthritis, mesenchymal stromal cells, secretome, extracellular vesicles, miRNAs, immune cells, cartilage

## Abstract

**Simple Summary:**

Osteoarthritis (OA) is a common and disabling condition affecting half billion people worldwide. To date, management has been only palliative and regenerative approaches based on mesenchymal stromal cells (MSCs) have therefore gained interest in the scientific and medical communities. One of the main obstacles for their translation into everyday practice is the lack of a thorough characterization of these biological products in the context of the disease. In this study, we aimed at dissecting the molecular signals released by MSCs in an environment resembling OA joints. The main findings are that MSCs in contact with synovial fluid of OA patients are able to release factors with anti-inflammatory and regenerative properties. These results will lay the foundations for a wider use of MSCs based products for regenerative medicine and hopefully slow down or halt progression of the disease in patients that, when pharmacological, biologic or surgical treatments fail, have prosthesis implant as the elective therapeutic option.

**Abstract:**

Bone marrow-derived mesenchymal stromal cells (BMSCs)-based therapies show a great potential to manage inflammation and tissue degeneration in osteoarthritis (OA) patients. Clinical trials showed the ability to manage pain and activation of immune cells and allowed restoration of damaged cartilage. To date, a molecular fingerprint of BMSC-secreted molecules in OA joint conditions able to support clinical outcomes is missing; the lack of that molecular bridge between BMSC activity and clinical results hampers clinical awareness and translation into practice. In this study, BMSCs were cultured in synovial fluid (SF) obtained from OA patients and, for the first time, a thorough characterization of soluble factors and extracellular vesicles (EVs)-embedded miRNAs was performed in this condition. Molecular data were sifted through the sieve of molecules and pathways characterizing the OA phenotype in immune cells and joint tissues. One-hundred and twenty-five secreted factors and one-hundred and ninety-two miRNAs were identified. The combined action of both types of molecules was shown to, first, foster BMSCs interaction with the most important OA immune cells, such as macrophages and T cells, driving their switch towards an anti-inflammatory phenotype and, second, promote cartilage homeostasis assisting chondrocyte proliferation and attenuating the imbalance between destructive and protective extracellular matrix-related players. Overall, molecular data give an understanding of the clinical results observed in OA patients and can enable a faster translation of BMSC-based products into everyday clinical practice.

## 1. Introduction

Osteoarthritis (OA) is a common and disabling condition affecting 250 million people worldwide [1]. It is a complex chronic disease characterized by structural alterations in the hyaline articular cartilage [2] and the synovium [3], with further involvement of the entire set of joint elements such as subchondral bone, ligaments, capsule, and periarticular muscles [4]. The crucial feature of OA is an imbalance between the repair and destruction of joint tissues, involving the presence of an immune response mainly mediated by macrophages [5] and T cells [6] in the synovial membrane and fluid. Regarding macrophages, high levels are detected in OA patients compared to healthy controls and correlate with clinical symptoms [7]. Furthermore, increases in inflammatory macrophage associated molecules in OA patient synovial fluid (SF) are linked with clinical outcomes in OA [8,9]. Similarly, T cells in the SF are associated with the pathogenesis of OA [10] and are the major constituents of synovial infiltrates in the membranes of OA patients [11]. To date, no drugs have a disease-modifying effect on OA, but rather their action is mostly related to relief of symptoms [12]. For this reason, treatments that may reduce disease manifestation in addition to slowing or stopping its progression are actively sought. In this frame, mesenchymal stromal cells (MSCs) have emerged as an attractive option, both as GMP-grade products [13] and as point-of-care products [14] such as bone marrow aspirate concentrate (BMAC) [15], micro-fragmented adipose tissue (MFAT) [16] or stromal vascular fraction (SVF) [17].

In all these applications, MSCs showed the potential of tissue restoration and inflammation management [18], with these features mainly ascribed to their secreted factors, either free [19] or conveyed within extracellular vesicles (EVs) [20], such as miRNAs, altogether defining the cell “secretome” [21]. Thus, a comprehensive fingerprint of the MSC-secretome is mandatory to give a molecular explanation of MSC therapeutic benefits and allow a faster translation of these innovative medicinal options. In this perspective, the main pitfall of traditional analyses of secreted factors and EV-miRNAs is the culturing conditions that are far from those which MSCs encounter when injected in the knee of OA patients. Mostly, MSC-secretome analysis was conducted cultivating cells in standard medium [22]. Although giving valuable hints, only a pathological environment may drive a more reliable secretome fingerprint, as demonstrated for bone marrow-MSCs (BMSCs) exposed to both healthy and degenerative human intervertebral disc conditions [23]. This pivotal experiment showed that a pathological environment induced in BMSCs a pronounced secretory phenotype able to promote immunomodulation, adjustment of ECM synthesis and degradation imbalance, and ECM reorganization.

To shed light on the secretory capacity of BMSCs as a treatment for OA, the aim of this work was to mimic an osteoarthritic joint environment by cultivating cells in the presence of SF from OA patients and evaluate the presence and amount of both soluble factors and EV-embedded miRNAs. Identified molecules were discussed within the framework of OA affected cell and tissue types including cartilage, synovium, macrophages and T cells.

## 2. Materials and Methods

### 2.1. Synovial Fluid Collection

An aliquot of synovial fluid (SF) (mean volume 2.7 mL ± 2.2) was collected from 13 patients (8 females, 5 males, mean age 69 ± 8 years; Kellgren and Lawrence III–IV grade) undergoing total knee arthroplasty. Floating cells and debris were removed by centrifugation (16,000× *g*, 10 min at RT) and supernatants stored at −80 °C. Before experiments were performed, single aliquots were pooled.

### 2.2. ELISA Characterization of Pooled SF

Two hundred-fifty μL pooled SF were treated with 12.5 μL of 40 mg/mL Hyaluronidase (Sigma-Aldrich, Milan, Italy) and 1.25 μL of Protease Inhibitor Cocktail (abcam, Cambridge, MA, USA). Incubation was performed for 30 min at 37 °C. Two-fold diluted digested OA-SF was used for soluble factors detection with the enzyme-linked immunosorbent assay (ELISA) Quantibody^®^ Human Cytokine Array 4000 Kit (RayBiotech, Norcross, GA, USA, https://www.raybiotech.com/quantibody-human-cytokine-array-4000/, accessed on 3 October 2022) following manufacturer’s protocol and four technical replicates. Concentrations were determined by comparison with standard samples. The amount of each factor was determined as pg/mL.

### 2.3. Bone Marrow Collection, BMSCs Isolation and Expansion

Total bone marrow aspirate from 3 female donors (mean age 50 ± 2 years) was seeded at a concentration of 50,000 nucleated cells/cm^2^ in αMEM (Thermo Fisher Scientific, Waltham, MA, USA) supplemented with 10% FBS at 37 °C, 5% CO_2_, and 95% humidity. Colonies appearing after 2 weeks were detached and BMSCs seeded at 4000/cm^2^ and cultured up to passage 3 for all described experiments. For secretome collection, BMSCs at 70% confluence were cultured for 2 days in the presence of 50% pooled SF (1:1 diluted in complete cell culture medium). This concentration was used since in clinical trials BMSCs are usually suspended in a volume between 5 and 8 mL [24,25,26] and injected in the OA synovial cavity that was reported to contain between 3 and 11 mL of SF [27,28], depending on the cohort of patients, for a final 50% SF concentration after BMSCs administration. After the treatment, BMSCs were washed 3 times with PBS and serum-free αMEM added (0.07 mL/cm^2^). After 2 days, the secretome was collected and centrifuged at 376× *g* for 5 min at 4 °C, 1000× *g* for 15 min at 4 °C, 2000× *g* for 15 min at 4 °C and twice at 4000× *g* for 15 min at 4 °C. Clarified secretomes were used for ELISA and EVs analyses. After secretome removal, BMSCs were counted and viability assessed with a NucleoCounter NC-3000 (ChemoMetec, Allerod, Denmark).

### 2.4. Flow Cytometry Characterization of SF-Treated BMSCs

After 2 days in 50% pooled SF, BMSCs were detached and stained (30 min at 4 °C in the dark) with both hemato/endothelial (CD31-PerCP Vio700 clone REA730, CD34-FITC clone AC136, CD45-PE Vio770 clone REA747) and MSC (CD44-PE Vio770 clone REA690, CD73-PE clone REA804, CD90-FITC clone REA897, CD105-PerCP Vio700 clone REA794, CD271-PE clone REA844) markers (Miltenyi Biotec, Bergisch Gladbach, Germany). BMSCs were detected by flow cytometry with a CytoFLEX flow cytometer (Beckman Coulter, Fullerton, CA, USA), collecting a minimum of 30,000 events. The following combinations of antibodies were used: CD73/90/105/44 and CD34/271/31/45.

### 2.5. ELISA Characterization of SF-Treated BMSCs Secretome

The enzyme-linked immunosorbent assay (ELISA) Quantibody^®^ Human Cytokine Array 4000 Kit was used, as previously described, to assay 2-fold diluted 250 μL secretomes from SF-treated BMSCs. The amount of each factor in pg/mL was converted into pg/million cells by multiplying the original value for the total volume in ml and dividing by the total number of cells. Values are shown as mean ± SD.

### 2.6. Protein-Protein Interaction Network

The online tool STRING (http://www.string-db.org, accessed on 14 June 2022) (database v11.5) was used to generate interactome maps of ELISA-identified proteins. The following settings were used: (i) organism, *Homo sapiens*; (ii) meaning of network edges, evidence; (iii) active interaction sources, experiments; (iv) minimum required interaction scores, low confidence (0.150).

### 2.7. Characterization of EVs in SF-Treated BMSCs Secretomes

All analyses were performed after 1:1 secretome dilution with PBS.

Nanoparticle tracking analysis (NTA): secretomes were run by Nanosight NS-300 system (NanoSight Ltd., Amesbury, UK) (5 recordings of 60 s) and EVs visualized with NTA software v3.4 providing both high-resolution particle size distribution profiles and concentration measurements.

Flow cytometry: 3 aliquots were analyzed. (i) Unstained, (ii) 5(6)-carboxyfluorescein diacetate succinimidyl ester (CFDA-SE, Sigma-Aldrich, St. Louis, MO, USA) stained (1 µM final concentration, 30 min at 37 °C) to visualize EVs after transformation into FITC-channel fluorescent carboxyfluorescein succinimidyl ester (CFSE), iii) after CFDA-SE supplementation, stained (30 min at 4 °C) with CD9-APC clone HI9A, CD63-APC clone H5C6, CD81-APC clone 5A6, CD44-APC clone BJ18, CD73-APC clone AD2, CD90-APC clone 5E10 (Biolegend, San Die-go, CA, USA). Samples were analyzed with a CytoFlex flow cytometer collecting at least 30,000 events. FITC-fluorescent nanobeads (160, 200, 240, and 500 nm, Biocytex, Marseille, France) were used as internal control for efficient detection in the nanometric range.

### 2.8. Total RNA Isolation from EVs and miRNAs Quantification

Secretomes were 1:1 diluted in PBS for a total volume of 10 mL and ultra centrifugated (100,000× *g*, 9 h at 4 °C) in an Optima L-90K Ultracentrifuge (Beckman Coulter) equipped with a Type 70.1 Ti Fixed-Angle Titanium Rotor (Beckman Coulter). To evaluate the efficiency of RNA recovery and cDNA synthesis, an exogenous *Arabidopsis thaliana* ath-miR-159a (30 pg) synthetic miRNA spike was added to EV pellets before total RNA extraction with miRNeasy and RNeasy Cleanup Kits (Qiagen, Hilden, Germany). The OpenArray system (Life Technologies, Foster City, CA, USA) equipped with the 384-well OpenArray plates was used to detect the presence of 754 miRNAs, according to the manufacturer’s instructions. Each single miRNA was considered as present and considered for further analyses only when amplification appeared in all three samples. The equalization of technical differences was performed scoring ath-miR-159 spike-in C_RT._ Eventually, the global mean method allowed normalization between samples.

### 2.9. Identification of miRNAs Target

The mRNA targets of detected miRNAs were identified with miRTarBase v8.0 (https://mirtarbase.cuhk.edu.cn/~miRTarBase/miRTarBase_2022/php/index.php, accessed on 14 March 2022) [29]. Only miRNA-mRNA interactions supported by strong experimental evidence were considered.

### 2.10. Statistical and Computational Analyses

Statistical analyses were performed with GraphPad Prism Software version 5 (GraphPad, San Diego, CA, USA). The Pearson correlation coefficient (R^2^) formula was used to calculate the linear association between samples. The outcome results were interpreted according to the degree of association [30].

Principal component analysis (PCA) and hierarchical clustering were obtained with ClustVis package (https://biit.cs.ut.ee/clustvis/, accessed on 15 June 2022) [31]. Maps were generated using the following settings for both rows and columns clustering distance and method: correlation and average, respectively.

## 3. Results

### 3.1. SF Characterization

The values of 190 detected soluble factors in pooled SF are shown in Supplementary Table S1. Three molecules were detected at >100,000 pg/mL: Insulin-like growth factor-binding protein 4 (IGFBP4, 248,050), Vascular cell adhesion protein 1 (VCAM1, 109,797) and Intercellular adhesion molecule 2 (ICAM2, 109,414). Other IGFBPs found at high levels, included IGFBP2 (44,358), IGFBP3 (42,150), IGFBP6 (6732) and IGFBP1 (5671). Out of classically described OA-related inflammatory cytokines, IL1B (99), IFNG (86) and IL6 (209) were found, while TNFA was absent. The recently described and synovia pro-inflammatory Interferon lambda-1 (IFNL1) was found at a high level (13,977), with Interferon lambda-2 (IFNL2) at a lower level (274). TGFB1, an OA-SF reported molecule, was amongst the most abundant molecules (29,154) as well as Plasminogen (PLG, 42,376) and its activator Urokinase plasminogen activator surface receptor (PLAUR, 11,597) along with PLAUR repressor Plasminogen activator inhibitor 1 (SERPINE1, 7097). Of note, 3 molecules interfering with IL1B signaling were detected: Interleukin-1 receptor antagonist protein (IL1RN, 459), Interleukin-1 receptor-like 1 (IL1RL1, 182) and Interleukin-1 receptor type 1 (IL1R1, 9). With a similar function on IL6, Interleukin-6 receptor subunit alpha (IL6R, 19,933) and Interleukin-6 receptor subunit beta (IL6ST, 15,002) were found at high levels. Although TNFA was not present, several receptors could be identified, including Tumor necrosis factor receptor superfamily member 1A (TNFRSF1A, 19,497), 17 (12,355), 1B (10,977), 14 (2201), 21 (1902), 10C (993), 18 (404), 10D (313), 9 (263), 8 (228) and 11B (104). Finally, 2 inhibitors of ECM-degrading enzymes were strongly detected: Metalloproteinase inhibitor 2 (TIMP2, 25,220) and 1 (TIMP1, 9879). The >10,000 pg/mL factors are shown in Table 1.

### 3.2. Immunophenotype of SF-Treated BMSCs

**Figure 1:**
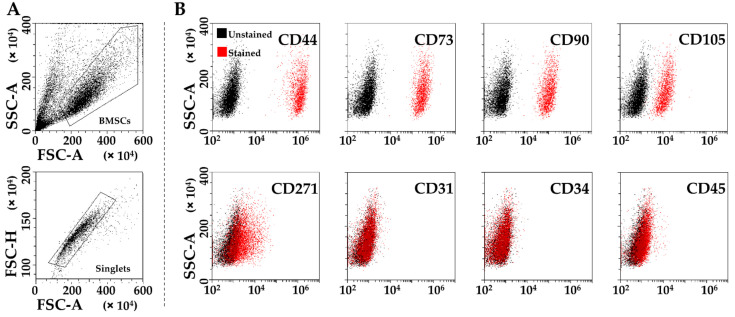
Flow cytometry analysis of SF-treated BMSCs. (**A**), after exclusion of debris (upper panel), single cells were identified (lower panel). (**B**), staining of single cells for general mesenchymal (CD44, CD73, CD90 and CD105, positive), BMSC-specific (CD271, positive) and hemato-endothelial markers (CD31, CD34, and CD45, negative). Representative plots are shown.

After 48 h in SF, BMSCs resulted strongly positive for MSC (CD44 100% ± 0, CD73 100% ± 0, CD 90 100% ± 0, CD105 95 % ± 4) and negative for hemato-endothelial (CD31 3% ± 0, CD34 0% ± 0, CD45 3% ± 1) markers (Figure 1). BMSCs were also positive to adult-MSC specific CD271 (19% ± 11), although at lower levels as previously reported [32] (Figure 1).

### 3.3. Characterization of SF-Treated BMSCs Secreted Factors

**Figure 2:**
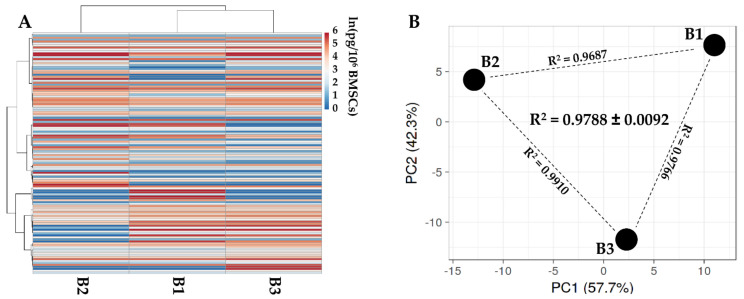
Comparison of secreted factor profiles between SF-treated BMSCs under study. (**A**), heat map of hierarchical clustering analysis of the ln(x) transformed pg/million BMSCs values of detected factors with sample clustering tree at the top. The color scale reflects the absolute expression levels: red shades = high expression levels and blue shades = low expression levels. B1, B2 and B3 stands for BMSC donors 1, 2 and 3. (**B**), principal component analysis of the ln(x) transformed pg/million BMSCs values of detected factors. X and Y axes show principal component 1 and principal component 2, which explain 57.7% and 42.3% of the total variance. B1, B2 and B3 stands for BMSC donors 1, 2 and 3.

One-hundred and twenty-five secreted factors were detected in all 3 SF-treated BMSCs secretomes (Supplementary Table S2). Hierarchical clustering showed a closer relationship between BMSCs 1 and 3, although PCA analysis highlighted a conserved distance between donors, further confirmed by correlation analysis (Figure 2). For these reasons, an average value was calculated for each factor. The 2 most abundant (>100,000 pg/million BMSCs) factors were IGFBP4 and 3. Another 9 molecules had an amount between 100,000 and 10,000 pg/million, including IGFBP2, TIMP1 and TIMP2, along with another ECM protective factor (SERPINE1). In this group, Transforming growth factor beta-1 (TGFB1), IFNL1, Interleukin-9 (IL9), Bone morphogenetic protein 4 (BMP4) and Vascular endothelial growth factor A (VEGFA) were also found. Twenty-four factors had a concentration between 10,000 and 1000 pg/million, with Platelet factor 4 (PF4) and IGFBP6 being by far the most abundant ones. In this group, C-C and C-X-C motif chemokines appeared, including CCL21/26/27 and CXCL11/16. A few growth factors could be found, such as Hepatocyte growth factor-like protein (MST1), Hepatocyte growth factor (HGF) and Fibroblast growth factor 4 (FGF4). Forty-eight proteins populated the 1000 to 100 pg/million group, with 2 C-C motif chemokines being among the most abundant (CCL2/25) along with other C-C and C-X-C motif factors (CCL5 and CXCL9/10/12). In this group, several interleukins were also present including IL1A/6/16/17B/23A/31 and IL8 (also known as CXCL8). C-C/C-X-C chemokines and interleukins also populated the <100 pg/million group of 42 factors, in particular, CCL1/4/7/8/11/13/14/16/17/18/20/24 and CXCL5/13, with IL1B/2/4/7/12/15. In this group, many growth factors could also be found, including Protransforming growth factor alpha (TGFA), Placenta growth factor (PGF), Granulocyte/macrophage colony-stimulating factor (CSF1/2/3), Platelet-derived growth factor subunit A (PDGFA), Brain-derived neurotrophic factor (BDNF), Beta-nerve growth factor (NGF) and Pro-epidermal growth factor (EGF). IFNG was also in this group. The most abundant factors (>1000 pg/million BMSCs) are shown in Table 2.

**Figure 3:**
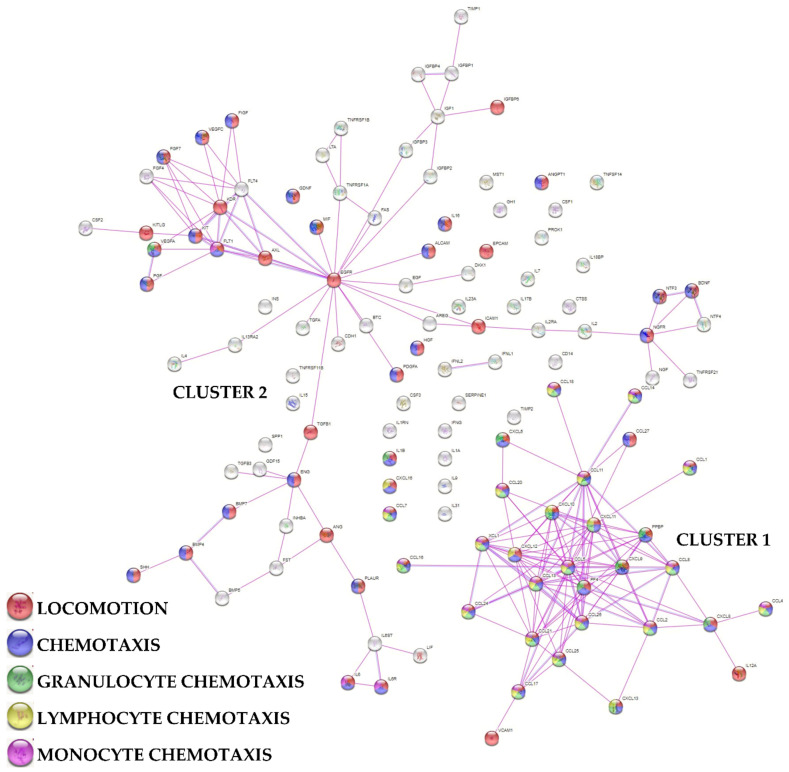
Functional association network for identified secreted factors released by SF-treated BMSCs. Using the online tool STRING, protein-protein interaction levels for 125 proteins of the BMSCs secretome were mined. The blue connections are for proteins with known interactions based on curated databases; violet connections for proteins with experimentally determined interactions. Empty nodes, proteins of unknown 3D structure; filled nodes, known or predicted 3D structure. Locomotion, chemotaxis, granulocyte chemotaxis, lymphocyte chemotaxis and monocyte chemotaxis related factors are shown.

**Figure 4:**
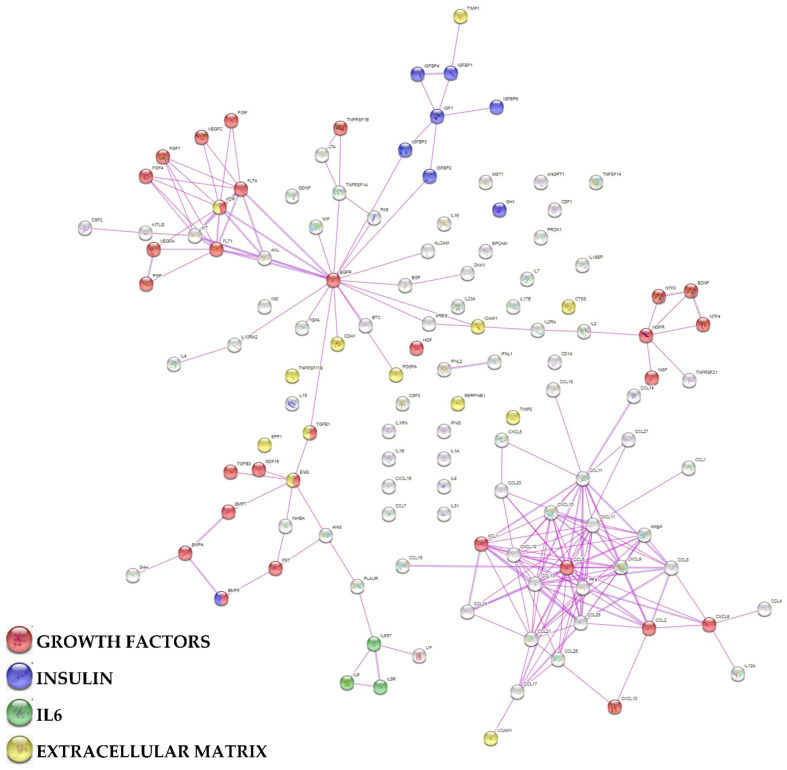
Functional association network for identified secreted factors released by SF-treated BMSCs. Using the online tool STRING, protein-protein interaction levels for 125 proteins of the BMSCs secretome were mined. The blue connections are for proteins with known interactions based on curated databases; violet connections for proteins with experimentally determined interactions. Empty nodes, proteins of unknown 3D structure; filled nodes, known or predicted 3D structure. Growth factors, insulin, IL6 and extracellular matrix related factors are shown.

To assign an overall function to the detected factors, a protein association network analysis was performed (Figure 3 and Figure 4). Two main clusters emerged, one tighter (Cluster 1) and another spread along the interaction map. In particular, Cluster 1 was composed of 28 proteins, all belonging to the gene ontology (GO) groups Locomotion (GO:0040011; see Supplementary Table S3 for factors related to this term and others in the text below) and Chemotaxis (GO:0006935) with the exception of IL12A and VCAM1 for this term (Figure 3). After sifting through the different blood cell types, 24 factors were related to Granulocytes (GO:0071621), 20 to Lymphocytes (GO:0048247) and 16 to Monocytes (GO:0002548). Locomotion and Chemotaxis also defined a small subgroup within Cluster 2 irradiating from EGFR. In this case, no obvious distinction of further levels related to blood cell types was observed. Notably, in this subgroup, several growth factors defined the term Cellular response to growth factor stimulus (GO:0071363). They were shared with 2 other small subgroups connected with EGFR, one defined by neuro-related proteins and one more heterogeneous with preponderance of BMPs (Figure 4), from which a small cluster associated with IL6 and its receptors (Interleukin-6 mediated signaling pathway, GO:0070102) could be identified. From EGFR, another small subcluster could be framed and associated with insulin signaling (Regulation of insulin-like growth factor receptor signaling pathway, GO:0043567). Finally, 13 proteins were related to Extracellular Matrix Organization (GO:0030198), without the formation of an interconnected cluster or group.

### 3.4. Characterization of SF-Treated BMSC-EVs

**Figure 5:**
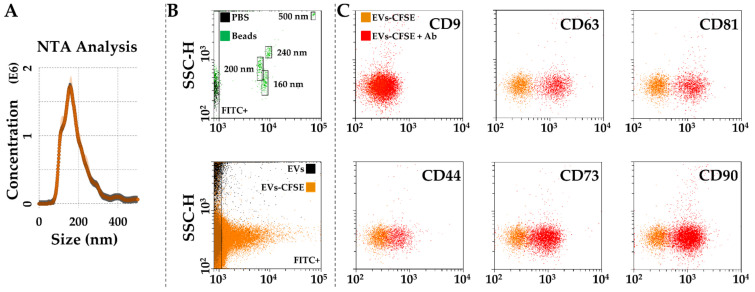
Characterization of SF-treated BMSC-EVs. (**A**), EVs size analysis from NTA data. (**B**), flow cytometer was first calibrated to score FITC-fluorescent particles of nanometer scale (upper panel, starting from 160 nm). EVs were CFSE stained to allow their identification and gating in the FITC channel (lower panel). (**C**), after gating, CFSE^+^ EVs showed positive staining for extracellular vesicle defining molecules CD63 and CD81, and MSC markers CD44, CD73 and CD90. CD9, another EV postulated marker, was barely detectable. Representative cytograms are presented.

After SF treatment, BMSCs released 537 ± 68 EVs per cell in 48 h. EVs had an average mode size of 155 nm ± 3, with 65 % of particles below 200 nm (Figure 5A). Flow cytometry analysis confirmed dimensional data after comparison with latex beads of nanometric size (Figure 5B). Particles were positive for CD63 (91% ± 0) and CD81 (90.3% ± 0.5) EV markers, while almost negative for CD9 (4.7% ± 0.5) as previously shown for BMSC-EVs [33] (Figure 5C). With respect to MSC-lineage markers, EVs were strongly positive for CD73 (82.3% ± 1.7) and CD90 (85.3% ± 0.5), while CD44 staining showed lower expression (46.0% ± 1) although the complete population shift suggested the homogeneous presence of the epitope (Figure 5C).

### 3.5. Identification of EV-Embedded miRNAs

**Figure 6:**
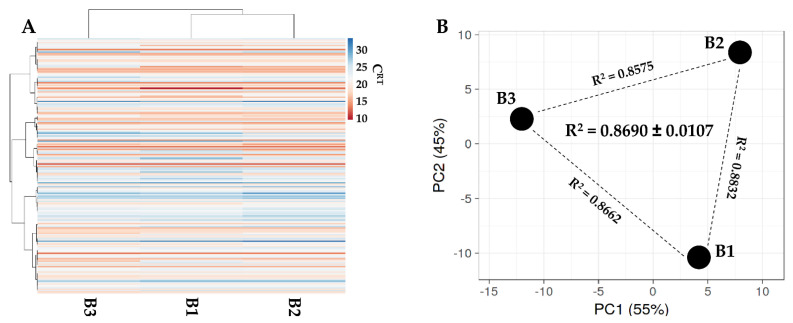
Comparison of EV-miRNA expression profiles between SF-treated BMSCs under study. (**A**), heat map of hierarchical clustering analysis of the normalized C_RT_ values of detected miRNAs with sample clustering tree at the top. The color scale reflects the absolute expression levels: red shades = high expression levels (low C_RT_ values) and blue shades = low expression levels (high C_RT_ values). B1, B2 and B3 stands for BMSC donors 1, 2 and 3. (**B**), principal component analysis of the normalized C_RT_ values of detected miRNAs. X and Y axis show principal component 1 and principal component 2 that explain 55% and 45% of the total variance. B1, B2 and B3 stands for BMSC donors 1, 2 and 3.

One-hundred and ninety-two miRNAs were identified at varying levels of intensity (Supplementary Table S4). Hierarchical clustering showed closer similitude for B1 and B2 samples (Figure 6A), although a pattern of overall similarity emerged by both PCA and correlation analyses (Figure 6B). For these reasons an average value for each miRNA was calculated. Moreover, to sharpen the genetic message, only miRNAs falling in the first quartile of expression were considered for further analyses. This choice is based on the knowledge that, even for the most abundant miRNAs, in MSC-EVs no more than one molecule per EV is present [34] and that to transfer one miRNA molecule to a recipient cell at least 100 EVs are needed [35]. Within this frame, 46 miRNAs emerged (Table 3) that were able to represent the 96.3% of the screened genetic weight of EVs. Further, to avoid possible misleading players, hsa-miR-720 and hsa-miR-1274A/B were excluded from analyses, being likely fragments of tRNA [36]. The most abundant miRNAs were hsa-miR-518f-3p (17.8% genetic weight), hsa-miR-24-3p (13.2%) and hsa-miR-193b-3p (8.39%). At the bottom of the quartile were hsa-miR-34a-5p (0.2%), hsa-miR-376a-3p (0.2%) and hsa-miR186-5p (0.2%). To evaluate EV-miRNAs’ effect in recipient cells, experimentally validated miRNA-mRNA interactions were sifted (Supplementary Table S5). miRNAs with the highest number of reported interactions were hsa-miR-145-5p (1.25%, 143 mRNA targets), hsa-miR-21-5p (0.88%, 135) and hsa-miR-34-5p (0.17%, 132). Analyzing the top 3 most abundant miRNAs, the interactions were: hsa-miR-518f-3p (17.76%, 0 targets), hsa-miR-24-3p (13.17%, 88) and hsa-miR-193b-3p (8.39%, 17). Considering all first quartile miRNAs, 1142 univocal mRNAs were validated (Supplementary Table S6).

### 3.6. EV-miRNAs Effect on OA-Related Molecules and Cell Types

To envision the effect of SF-treated BMSC-EVs miRNAs for OA pathology, reported OA-regulators expressed in at least 1% of OA chondrocytes, synoviocytes, macrophages and T cells [37] were compared with the list of first quartile miRNA targets (Table 4). Of note, the majority of pro-inflammatory cytokines reported to induce and sustain OA were targeted, including IL1A/B, IL6 and TNF although this last one was not detected in the OA synovial fluid used in the study. The most targeted (>1% of the scored EV genetic weight) cytokine was IL1A, due to hsa-miR-191-5p (5.58% of the genetic weight), followed by TNF (main regulator hsa-miR-125-5p, 1.23%) and CCL5 (main hsa-miR-214-3p, 1.32%). Concerning growth factors, TGFB1 was by far the most targeted molecule, due to the second most abundant miRNA hsa-miR-24-3p (13.2%) and hsa-miR574-3p (5.88%). Other preferential (>1% EV weight) targets were VEGFA, regulated by 10 miRNAs leaded by hsa-miR-145-5p (1.25%), KITLG (hsa-miR-320a-3p, 3.47%), ANGPT2 (main hsa-miR-145-5p, 1.25%), TGFB2 (main hsa-miR-145-5p, 1.25%), CTGF (main hsa-miR-145-5p, 1.25%), BMP2 (main hsa-miR-106a-5p, 0.83%), IGF2 (main hsa-miR-125b-5p, 1.23%), BDNF (main hsa-miR-16-5p, 0.82%) and HGF (main hsa-miR-16-5p, 0.82%). Looking at proteases and related factors, EV-miRNAs target 11 proteins involved with ECM degradation and only 2 with protective features. In the first group, the top factors were MMP14 (mainly due to hsa-miR-24-3p, 13.17%), MMP1 (main hsa-miR-222-3p, 7.59%) and PLAU (hsa-miR-193b-3p, 8.39%). Other proteases preferentially inhibited by EV-miRNAs were MMP2 (main hsa-miR-125b-5p, 1.23%), ADAM17 (hsa-miR-145-5p, 1.25%) and MMP13 (hsa-miR-125b-5p, 1.23%). For ECM protective factors, TIMP3 is the most heavily targeted (main hsa-miR-222-3p, 7.59%), followed by TIMP2 (main hsa-miR-106a-5p, 0.83%). Further, due to its abundance, the most impactful miRNA was hsa-miR-24-3p (13.17%), able to target both TGFB1 and MMP14. Other miRNAs that can tip the protection/destruction balance were hsa-miR-193b-3p (8.39%) regulating PLAU and hsa-miR-222-3p (7.59%) having a dual role by acting on MMP1 and TIMP3. Other miRNAs able to strongly regulate OA factors were hsa-miR-574-3p (5.88%, targets TGFB1), hsa-miR-191-5p (5.58%, IL1A) and hsa-miR320a-3p (3.47%, KITLG). Lastly, synoviocytes were the most targeted cells, with 32 OA-regulators, followed by HLA-DR+ cells (including inflammatory macrophages) (19), chondrocytes (17) and T cells (2).

Eventually, abundant miRNAs were sifted through available literature describing those miRNAs directly involved in homeostasis, inflammation, protection and destruction of the most important cell types driving OA (Table 5). Regarding cartilage [38], 9 miRNAs have protective functions and 7 have destructive functions. The overall genetic weight for the protective group is mostly due to 5 miRNAs with single weight >1% (hsa-miR-24-3p, 13.17%; hsa-miR-193b-3p, 8.39%; hsa-miR-222-3p, 7.59%, hsa-miR-320a-3p, 3.47%; hsa-miR-125b-5p, 1.23%), for a total of 35.94%. In contrast, none of the destructive miRNAs had a weight >1%, for a total of 3.10%. Therefore, overall, the protection vs. destruction ratio is 11.6 in favor of cartilage healing and maintenance. For synovia, to date, very little is known about the role of single miRNAs [39]. We identified one miRNA (hsa-miR-29a-3p, 0.64%) with protective and one (hsa-miR-34a-5p, 0.17%) with destructive properties, together with hsa-miR-146a-5p (0.19%) having a dual role. Thus, it is not possible to outline a clear picture. Concerning macrophages [40], 2 miRNAs were reported to promote a pro-inflammatory M1 while 5 miRNAs supported an anti-inflammatory M2 phenotype. The overall weight was 2.48% for inflammation (hsa-miR-145-5p, 1.25%; hsa-miR-125b-5p, 1.23%) vs. 21.83% for anti-inflammation (mainly due to hsa-miR24-3p, 13.17% and hsa-miR-222-3p, 7.59%), for a ratio of 8.8 in favor of M2 macrophages. Finally, miRNAs involved in T cells [41] were analyzed. Eight pro-activating and 4 anti-activating miRNAs were found. Due to hsa-miR-24-3p (13.17%), repressing miRNAs had a total weight of 15.48% vs. 6.88% of activating molecules, ending in a ratio of 2.3 in favor of inhibition of T cell activity. Thus, overall, protection and inflammation reduction signals far exceeded damaging and pro-inflammatory features for almost all OA-affected tissues under analysis.

## 4. Discussion

In this report the secreted factors and EV-embedded miRNAs released by BMSCs treated with synovial fluid of OA patients were analyzed for the first time. The main strength and innovative feature of this study lies in mimicking a closer therapeutic application of BMSC-derived products. In fact, the exposure of BMSC to the synovial fluid of patients with OA instead of single pro-inflammatory molecules makes the observations more reliable and similar to what happens in the clinical setting.

The main findings were that the soluble molecules released by BMSCs in the presence of OA synovial fluid are involved with locomotion and chemotaxis of immune cells while EV-miRNAs have protective and anti-inflammatory roles in the tissues and cells, promoting and maintaining the OA phenotype.

In recent years, several clinical studies have investigated BMSC-based therapies for OA [42], including both randomized control trials and observational studies. Most of the reports included patients with grade II–III of Kellgren–Lawrence, with some cases of grade IV suggesting that the therapy may be envisioned to be applied for both early and late stage OA. The main finding of these studies was the improvement of the function of the knee joint after BMSCs intra-articular injections, along with gain in tissue structure and overall pain reduction. Regarding cartilage, a few studies also reported a decrease of poor cartilage areas with tissue quality improvement quantified by radiographic and MRI measurements [24,25,43,44], although in small patient cohorts. BMSCs also reduced pain [26,44,45,46], with results, maintained up to several months, that allowed an increase in the walking time function [26]. BMSCs injections were also shown to relieve synovitis with a concomitant decrease of pro-inflammatory macrophages in synovial fluids [47]. For these reasons, BMSC-based therapies showed a sufficient effect to postpone or avoid knee replacement when studied in the contra-lateral joint in patients with bilateral osteoarthritis [48].

These anti-inflammatory and protective/regenerative results observed in OA patients may find a molecular background by the properties of the soluble factors and EV-miRNAs in the secretome of BMSCs exposed to OA synovial fluid, as described in this paper. Regarding inflammation, BMSCs were reported to interact with many kinds of immune cells, including macrophages [49,50] and T cells [50,51], with cell-cell contact being postulated to facilitate MSC-regulated immunosuppression [52]. In this view, SF-treated BMSCs secrete several molecules able to attract all immune cells, including macrophages and lymphocytes. Once in proximity, BMSCs may promote phenotype switch through factors and EV-miRNAs. In the first group, we identified IL1RN (alias IL1RA, found at 280 pg/mL), a well described molecule mediating the MSCs immunosuppressive effect at different levels. IL1RA was shown to inhibit the proliferation of T lymphocytes, to increase the amount of Tregs and to induce the macrophage polarization from M1 to M2 phenotype, in turn secreting IL10 and exerting an additional suppressive effect on T cells [53]. Moreover, in a collagen-induced OA murine model, IL1RA was a crucial factor in protection from OA progression by decreasing the percentage of activated T lymphocytes and increasing the percentage of Tregs [53]. A similar effect on Tregs was proposed for other two molecules present in SF-treated BMSCs secretome, CCL18 (46 pg/mL) and TGFB1 (34,048 pg/mL), with their neutralization leading to a significant reduction in MSC-induced Tregs formation from conventional T cells [54,55]. Of note, both molecules are among the drivers of the formation of FoxP3+ Tregs from naïve CD4+ T cell [54,56]. BMSC-secreted TGFB1 was also reported to suppress T cell proliferation [57] and, on a more general level, was shown to prevent their activation [58]. Similarly, BMSC-derived ICAM1 (178 pg/mL) was demonstrated to be critical for the MSC-mediated immunosuppression of T cells, contributing to the rapid suppression of TNF and IFNG in activated T cells [59]. These effects on immune cells for factors released after OA-SF treatment were supported by the function of the most abundant EV-embedded miRNAs, which target several well described OA inflammatory cytokines such as IL1A/B, IL6 and TNF. EV-miRNAs also target other important cytokines associated with OA, such as CXCL12, whose levels in SF were closely related to the radiographic severity of OA [60], CCL5, recruiting Th1/17/22 to the affected joint triggering the inflammation process [61], and IL11, recently shown to be upregulated with OA severity in the synovial fluid of OA patients [62]. Of importance, all these targeted inflammatory cytokines are expressed by OA HLA-DR+ cells, including M1 macrophages, and activated T cells for CCL5. Consistently, overall, the most abundant miRNAs have a preponderance for M2 polarization of macrophages and for reduction of activation of T cells. For both cell types, the balance towards immune suppression is driven by hsa-miR-24-3p (13.17 % of the EV genetic weight). This miRNA was shown to regulate macrophage polarization and plasticity, inhibiting M1 and supporting M2 phenotype when overexpressed [63], with basal hsa-miR-24-3p expression being higher in M2 macrophages [64]. For T cells, hsa-miR-24-3p targets IFNG in activated CD4+ [65] and CD8+ [66] cells, with its delivery through EVs being able to inhibit T-cell proliferation and Th1 and Th17 differentiation and induce Tregs [67]. Finally, another EV-miRNA with an important role in tipping the balance towards macrophage M2 polarization is hsa-miR-22-3p (7.59 %), whose expression is again opposed to IFNG [68]. Thus, overall, the combination of soluble factors and EV-miRNAs support the observed anti-inflammatory effects of BMSCs when injected in the OA joint through a combined action relying on SF immune cell attraction followed by phenotype polarization, especially for macrophages and T cells. The intrinsic capacity of secreted molecules and EVs to permeate the synovia [69] suggest that their modulatory properties can be envisioned also for tissue resident immune cells that greatly contribute to OA joint inflammatory status [70].

Together with immune cell interaction, secreted molecules and EV-miRNAs suggested a molecular background for tissue protection, with particular focus on cartilage. Among the most abundant secreted factors, TIMP1 (13,727 pg/mL) and TIMP2 (26,963 pg/mL) were widely reported as crucial molecules for cartilage protection. TIMPs are key regulators of the metalloproteinases (MMPs) that degrade the extracellular matrix (ECM) [71], this phenomenon being one of the main events leading to cartilage disruption in OA. Accordingly, the supplementation of bovine cartilage with TIMP1/2 prevented the release of collagen fragments [72], and new molecules altering pathological MMPs/TIMPs imbalance in OA joints are currently being tested. An example is Paeoniflorin, a pinane monoterpene glucoside, that was able to downregulate the expression of MMPs and increase the expression of TIMP1 mRNA and protein in rat chondrocytes [73]. A second example is the histone deacetylase inhibitor trichostatin A that in OA rats was able to reduce the imbalance of the TIMPs/MMPs ratio through the increase of TIMP1 and decrease of MMP1/3/13 [74]. In this frame, injection of BMSCs could be a way to tip the balance towards protection through the release of TIMP1 and 2. Further, among the most abundantly secreted molecules, TGFB1 (34,048 pg/mL) was also proposed as an OA therapeutic tool. In fact, TGFB1 is crucial for cartilage maintenance and its supplementation can enhance cartilage repair [75]. Nevertheless, it may also create problems in other tissues of the joint like fibrosis of the synovia and osteophyte formation. In this regard, SF-treated BMSCs might be an intriguing option, due to the concurrent release of both TGFB1 and EV-embedded miRNAs such as hsa-miR-24-3p that can locally inhibit TGFB synthesis at sites of unwanted side effects such as bone/cartilage and synovia. In the same paradigm of BMSCs secreted molecules as therapeutic tools, SerpinE1 (12,649 pg/mL) is a direct inhibitor of both tissue- and urokinase-type activators of plasminogen into plasmin, thereby regulating plasmin-related cleavage of ECM components such as fibronectin, glycoproteins and proteoglycans and direct activation of MMPs [76]. Consistently, SerpinE1 was able to protect against cartilage collagen breakdown in an ex vivo model of cartilage destruction through the inhibition of proteolytic activators of MMPs, with its levels decreasing in OA cartilage together with an overall increase of plasmin activity [77]. Again, under the paradigm of secretome molecules as part of the therapeutic tool, the abundant BMP4 (12,946 pg/mL) plays a crucial role in maintaining a chondrogenic phenotype and enhancing matrix production [78]. Consistently, BMP4 (12,946 pg/mL) in association with muscle MSCs, was able to efficiently regenerate cartilage when injected intra-articularly in a rat OA model [79], and BMP4-transfected adipose-MSCs significantly improved in vivo chondrogenesis in a rabbit OA model [80]. Thus, several SF-treated BMSCs secretome molecules have ECM and cartilage protective roles. Nevertheless, there are some abundant factors that might have a tricky effect on cartilage and ECM. Some falling in this category are IGFBPs, including the 2 most detected proteins (IGFBP4, 122,009 pg/mL and IGFBP3, 101,105 pg/mL) and other members of the family (IGFBP2, 10,410 pg/mL, IGFBP6, 8763 pg/mL and IGFBP1, 724 pg/mL). They bind IGFs, known to have potent anabolic actions on chondrocytes. Since they are increased in OA cartilage, IGFBPs’ binding and masking of IGF1 might underlie the reduced cartilage matrix synthesis in degenerated areas of OA cartilage [81], making IGFBPs detrimental factors. Nevertheless, in cartilage, ECM macromolecular complexes between IGFBPs and IGFs have been detected that, when degraded by MMPs acting on IGFBPs, lead to increased amounts of available IGFs around chondrocytes as part of an attempted repair process of the cartilage structure [82]. Of note, the ratio between cartilage ECM synthesis and degradation, that is strongly affected in OA, is not only regulated by inhibitors or other proteins influencing their amounts/activity but also by several EV-embedded miRNAs, making the picture far more complex. In fact, we observed that abundant miRNAs may target several MMPs, in particular MMP1 and 2, and other ECM degrading proteases such as ADAMs, PLAU/PLAT and APC, while only TIMP3 had strong targeting. In particular, the urokinase-type activator PLAU, that is increased in its levels in both OA-SF and cartilage [83], is heavily targeted by hsa-miR-193b-3p (8.39 %). This miRNA, together with hsa-miR-24-3p (13.17 %), miR-222-3p (7.59 %) and miR-320a-3p (3.47 %) is responsible for the overall cartilage protective role of EV-embedded miRNAs that go beyond the regulation of single proteins. hsa-miR-24-3p was reduced in OA cartilage; in chondrocytes, its overexpression downregulated apoptosis, inflammation and ECM degradation by targeting BCL2L12 [84]. hsa-miR-193b-3p expression was significantly reduced in OA cartilage and its overexpression strongly enhanced in vivo cartilage formation by directly targeting HDAC3 and promoting H3 acetylation [85]. hsa-miR-222-3p was found significantly downregulated in OA cartilage [86] and its over-expression significantly suppressed cartilage destruction by targeting HDAC4 [87]. Eventually, hsa-miR-320-3p expression was significantly reduced in OA cartilage and its overexpression was associated with increased collagen deposition and COL2A1 expression [88]. Therefore, overall, secreted factors and EV-miRNAs account for the cartilage protective features of BMSCs when injected intra-articularly in the joints of OA patients.

We are aware that this study has some limitations. First, the number of factors and miRNAs is limited to the techniques used, ELISA and qRT-PCR. Especially for miRNAs, we preferred to detect the presence of very well characterized players, being the vast majority of the, to date (September 2022), 38,589 identified molecules still lacking a proper characterization not only for OA but also in relation to other pathologies or regulated targets and pathways. Second, the secretome was obtained in starving conditions after culturing BMSCs in synovial fluid. This was necessary to avoid contamination of FBS and SF-derived factors and EVs. The OA-SF used in the study was obtained pooling several samples and using this pool on BMSCs obtained from a different set of patients. This choice was due to the reduced amount of SF that could be obtained from single patients. We are aware that the analyte quantities reported in Table 1 for the pooled SF are not necessarily reflective of the 13 single individual samples and that one sample could have been responsible for the majority of the presence of a particular molecule of interest. Nevertheless, we preferred to describe absolute values rather than relative abundance to give a general roadmap of the molecules that can be found in OA-SF, being conscious that this is of particular relevance for the herein described set of experiments and that with single SF both individual factors and BMSC general response could be at least in part differently modulated. Moreover, it was not possible to obtain bone marrow samples from the same patients undergoing SF collection before knee surgery for obvious ethical reasons, avoiding treatments not related with the surgical procedure. Nevertheless, the choice of a more “realistic” model to test the secretory ability of BMSCs when exposed to a trigger such as OA would make the findings very interesting and worthy of further investigation.

## 5. Conclusions

BMSCs treatment for OA patients has shown promising results for both inflammation management and cartilage restoration allowing for increased quality of life and a delay of total knee arthroplasty. Soluble factors and EV-miRNAs released from BMSCs cultured in the synovial fluid of OA patients had strong immunomodulatory and cartilage protecting potential. The combined action of both types of molecules is able to promote BMSC interaction with the most common immune cells involved in OA, such as macrophages and T cells, driving their switch towards an anti-inflammatory phenotype. Further, cartilage homeostasis is stimulated by assisting chondrocyte proliferation and attenuating the imbalance in destructive/protective extracellular matrix-related players. Altogether, these data give a molecular grounding to the clinical results and will be a fundamental milestone, allowing a faster translation of this cutting-edge approach into everyday clinical practice.

## Figures and Tables

**Table 1:** >10,000 pg/mL pooled SF factors.

TYPE	FACTOR	(pg/mL)	
GF	IGFBP4	248,050	Insulin-like growth factor-binding protein 4
REC	VCAM1	109,797	Vascular cell adhesion protein 1
CYT	ICAM2	109,414	Intercellular adhesion molecule 2
GF	IGFBP2	44,358	Insulin-like growth factor-binding protein 2
CYT	PLG	42,376	Plasminogen
GF	IGFBP3	42,150	Insulin-like growth factor-binding protein 3
CYT	SIGLEC5	32,371	Sialic acid-binding Ig-like lectin 5
GF	TGFB1	29,154	Transforming growth factor beta-1
INF	TIMP2	25,220	Metalloproteinase inhibitor 2
REC	SELL	20,525	L-selectin
INF	IL6R	19,933	Interleukin-6 receptor subunit alpha
GF	BMP4	19,550	Bone morphogenetic protein 4
INF	TNFRSF1A	19,497	Tumor necrosis factor receptor superfamily member 1A
GF	CSF1R	19,117	Macrophage colony-stimulating factor 1 receptor
CYT	IL6ST	15,002	Interleukin-6 receptor subunit beta
CHE	IFNL1	13,977	Interferon lambda-1
REC	TNFRSF17	12,355	Tumor necrosis factor receptor superfamily member 17
REC	PLAUR	11,597	Urokinase plasminogen activator surface receptor
INF	TNFRSF1B	10,977	Tumor necrosis factor receptor superfamily member 1B

CHE: Chemokine; CYT: Cytokine; GF: Growth factor; INF: Inflammation; REC: Receptor.

**Table 2:** Most abundant (>1000 pg/million cells) SF-treated BMSCs factors.

		pg/Million BMSCs					
TYPE	FACTOR	B1	B2	B3	MEAN	SD	FUNCTION
GF	IGFBP4	109,869	131,569	124,587	122,009	9045	Insulin-like growth factor-binding protein 4
GF	IGFBP3	84,091	117,198	102,025	101,105	13,532	Insulin-like growth factor-binding protein 3
GF	TGFB1	39,339	32,615	30,189	34,048	3870	Transforming growth factor beta-1
INF	TIMP2	22,096	28,331	30,462	26,963	3550	Metalloproteinase inhibitor 2
CHE	IFNL1	19,399	20,613	22,381	20,798	1225	Interferon lambda-1
CHE	IL9	1770	24,217	16,270	14,086	9293	Interleukin-9
INF	TIMP1	12,116	14,563	14,503	13,727	1140	Metalloproteinase inhibitor 1
GF	BMP4	7872	10,892	20,075	12,946	5190	Bone morphogenetic protein 4
CYT	SERPINE1	10,110	14,584	13,253	12,649	1876	Plasminogen activator inhibitor 1
GF	IGFBP2	6439	14,130	10,661	10,410	3145	Insulin-like growth factor-binding protein 2
GF	VEGFA	7794	12,442	10,732	10,323	1919	Vascular endothelial growth factor A
CHE	PF4	9652	10,111	8745	9503	567	Platelet factor 4
GF	IGFBP6	6466	10,314	9509	8763	1657	Insulin-like growth factor-binding protein 6
CHE	MIF	3604	4526	4973	4368	570	Macrophage migration inhibitory factor
REC	VCAM1	4131	5719	2852	4234	1173	Vascular cell adhesion protein 1
CYT	INHBA	4344	4350	3770	4155	272	Inhibin beta A chain
CHE	XCL1	2921	4047	5177	4048	921	Lymphotactin
CHE	CCL27	4283	2370	3979	3544	840	C-C motif chemokine 27
CHE	CXCL16	2374	3787	3932	3364	702	C-X-C motif chemokine 16
INF	TNFRSF1A	2640	3403	3410	3151	361	Tumor necrosis factor receptor superfamily member 1A
CHE	MST1	3356	1822	2150	2443	659	Hepatocyte growth factor-like protein
CHE	CCL26	2212	932	4108	2417	1305	C-C motif chemokine 26
CHE	CCL21	2552	3078	1594	2408	614	C-C motif chemokine 21
CHE	CXCL11	2165	3168	1466	2266	698	C-X-C motif chemokine 11
REC	PLAUR	1515	2482	1641	1879	429	Urokinase plasminogen activator surface receptor
CYT	ANGPT1	1553	2005	1837	1798	187	Angiopoietin-1
GF	BMP7	2239	327	1766	1444	813	Bone morphogenetic protein 7
CYT	IL6ST	1239	1842	1090	1390	325	Interleukin-6 receptor subunit beta
GF	HGF	1058	1462	1443	1321	186	Hepatocyte growth factor
GF	FGF4	1536	1484	729	1250	369	Fibroblast growth factor 4
CYT	ANG	971	1427	1301	1233	193	Angiogenin
REC	ALCAM	956	1209	1239	1135	127	CD166 antigen
CYT	FST	690	1260	1284	1078	274	Follistatin
CYT	CTSS	808	1256	1150	1071	191	Cathepsin S
GF	KDR	1078	887	1102	1022	96	Vascular endothelial growth factor receptor 2

CHE: Chemokine; CYT: Cytokine; GF: Growth factor; INF: Inflammation; REC: Receptor. B1, B2 and B3 stands for BMSC donors 1, 2 and 3.

**Table 3:** miRNAs detected in SF-treated BMSC-EVs and falling in the first quartile of expression.

miRBase ID	Crt					
	B1	B2	B3	Mean	SD	Weight %
hsa-miR-518f-3p	9.26	12.46	13.80	11.84	1.90	17.75728
hsa-miR-24-3p	12.47	12.14	12.20	12.27	0.15	13.16838
hsa-miR-193b-3p	13.04	12.78	12.95	12.92	0.11	8.388070
hsa-miR-222-3p	13.26	13.12	12.81	13.06	0.19	7.591257
*hsa-miR-1274B*	*13.12*	*13.18*	*13.33*	*13.21*	*0.09*	*6.863791*
hsa-miR-574-3p	13.50	13.24	13.55	13.43	0.14	5.882132
hsa-miR-191-5p	13.43	13.53	13.56	13.51	0.06	5.582864
hsa-miR-484	14.17	13.74	13.90	13.94	0.18	4.142033
hsa-miR-320a-3p	14.34	13.94	14.30	14.19	0.18	3.468564
hsa-miR-197-3p	15.20	14.51	14.89	14.86	0.28	2.181023
hsa-miR-19b-3p	15.44	15.55	15.68	15.55	0.10	1.352228
hsa-miR-214-3p	15.47	15.49	15.81	15.59	0.16	1.317989
hsa-miR-99a-5p	15.69	15.67	15.63	15.66	0.03	1.254117
hsa-miR-145-5p	15.61	15.59	15.80	15.67	0.09	1.250356
hsa-miR-125b-5p	15.76	15.67	15.65	15.69	0.05	1.226040
*hsa-miR-1274A*	*15.80*	*15.93*	*16.14*	*15.96*	*0.14*	*1.021016*
hsa-miR-627-5p	17.05	14.73	16.22	16.00	0.96	0.992408
hsa-miR-342-3p	16.22	15.80	16.38	16.13	0.25	0.904174
hsa-miR-409-3p	16.19	16.33	15.94	16.15	0.16	0.891932
hsa-miR-21-5p	15.80	16.24	16.46	16.17	0.28	0.884135
hsa-miR-106a-5p	16.02	16.42	16.33	16.25	0.17	0.832009
hsa-miR-16-5p	16.37	16.29	16.19	16.28	0.07	0.816961
hsa-miR-17-5p	16.10	16.36	16.39	16.28	0.13	0.815264
hsa-let-7b-5p	16.39	16.40	16.66	16.48	0.12	0.710056
hsa-miR-29a-3p	16.71	16.83	16.39	16.64	0.19	0.635518
hsa-miR-30c-5p	16.48	16.81	16.81	16.70	0.16	0.610052
hsa-miR-221-3p	16.79	16.75	16.86	16.80	0.05	0.569594
hsa-miR-92a-3p	17.12	16.93	17.10	17.05	0.08	0.478969
hsa-miR-30b-5p	16.93	17.20	17.35	17.16	0.17	0.444629
hsa-miR-20a-5p	16.92	17.28	17.43	17.21	0.21	0.428492
hsa-miR-132-3p	17.31	17.13	17.47	17.30	0.14	0.402206
hsa-miR-618	13.41	14.78	24.32	17.50	4.85	0.350464
hsa-miR-138-5p	17.39	17.43	17.87	17.56	0.22	0.335722
hsa-miR-382-5p	18.55	17.53	17.41	17.83	0.51	0.279323
hsa-miR-663b	17.74	17.68	18.19	17.87	0.23	0.271308
hsa-miR-483-5	18.29	17.46	17.99	17.91	0.34	0.264011
hsa-miR-199a-3p	18.14	18.30	17.86	18.10	0.18	0.231327
hsa-miR-520e-3p	15.88	17.10	21.67	18.22	2.49	0.213307
hsa-miR-31-5p	18.39	18.44	17.85	18.23	0.27	0.211883
hsa-miR-28-3p	18.39	18.51	18.18	18.36	0.14	0.193402
hsa-miR-146a-5p	18.47	18.77	18.02	18.42	0.31	0.185395
*hsa-miR-720*	*18.69*	*18.31*	*18.31*	*18.44*	*0.18*	*0.183435*
hsa-miR-193a-5p	18.27	18.37	18.71	18.45	0.19	0.181957
hsa-miR-34a-5p	19.15	18.58	17.84	18.52	0.54	0.172740
hsa-miR-376a-3p	18.51	18.59	18.58	18.56	0.03	0.168094
hsa-miR-186-5p	19.01	18.74	18.40	18.72	0.25	0.151005

In *italics* miRNAs that are possibly tRNA fragments and therefore excluded from analyses. B1, B2 and B3 stands for BMSC donors 1, 2 and 3.

**Table 4:** OA regulators expressed by at least 1% of indicated cell types and targeted by first quartile EV-miRNAs.

	CHO	SYN	HLA-DR+	T CELL	% WEIGHT	MAIN miRNA
CYTOKINES						
TNF		X	X		2.05	hsa-miR-125-5p (1.23%)
IL6		X	X		0.19	hsa-miR-146a-5p (0.19%)
IL1B		X	X		0.88	hsa-miR-21-5p (0.88%)
IL1A		X	X		5.58	hsa-miR-191-5p (5.58%)
CXCL12		X	X		0.97	hsa-miR-221-3p (0.57%)
CCL5		X	X	X	1.51	hsa-miR-214-3p (1.32%)
IL11	X	X	X		0.61	hsa-miR-30c-5p (0.61%)
**GROWTH** **FACTORS**						
TGFB1	X	X	X	X	19.24	hsa-miR-24-3p (13.17%)
IGF1		X	X		0.87	hsa-miR-29a-3p (0.64%)
FGF2	X	X			0.97	hsa-miR-16-5p (0.82%)
BMP2	X	X	X		1.65	hsa-miR-106a-5p (0.83%)
VEGFA	X	X	X		6.22	hsa-miR-145-5p (1.25%)
HGF		X	X		1.05	hsa-miR-16-5p (0.82%)
ANGPT2		X	X		2.48	hsa-miR-145-5p (1.25%)
CTGF	X	X	X		1.86	hsa-miR-145-5p (1.25%)
KITLG	X	X	X		3.47	hsa-miR-320a-3p (3.47%)
TGFB2	X	X	X		2.13	hsa-miR-145-5p (1.25%)
INHBB		X			0.17	hsa-miR-34a-5p (0.17%)
IGF2	X	X			1.5	hsa-miR-125b-5p (1.23%)
BDNF		X			1.22	hsa-miR-16-5p (0.82%)
**PROTEASES**						
ADAM12	X	X			0.64	hsa-miR-29a-3p (0.64%)
ADAM17	X	X	X		1.25	hsa-miR-145-5p (1.25%)
ADAMTS9		X			0.64	hsa-miR-29a-3p (0.64%)
MMP1		X			8.84	hsa-miR-222-3p (7.59%)
MMP2	X	X			3.26	hsa-miR-125b-5p (1.23%)
MMP9		X	X		0.4	hsa-miR-132-3p (0.40%)
MMP13					1.23	hsa-miR-125b-5p (1.23%)
MMP14	X	X			14.42	hsa-miR-24-3p (13.17%)
PLAU		X	X		8.39	hsa-miR-193b-3p (8.39%)
PLAT	X	X			0.88	hsa-miR-21-5p (0.88%)
APC	X	X			2.06	hsa-miR-125b-5p (1.23%)
TIMP2	X	X			1.26	hsa-miR-106a-5p (0.83%)
TIMP3	X	X			9.86	hsa-miR-222-3p (7.59%)

CHO stands for chondrocytes, SYN for synoviocytes.

**Table 5:** EV-miRNAs involved in homeostasis of OA-affected tissues and cells.

TISSUE/CELLS	% WEIGHT	ROLE
**CARTILAGE**		
*Protective*		
hsa-miR-24-3p	13.17	Prevents ECM degradation, increases chondrocyte viability
hsa-miR-193b-3p	8.39	Reduces cartilage degradation
hsa-miR-222-3p	7.59	Reduces cartilage degradation
hsa-miR-320a-3p	3.47	Increases chondrocyte viability
hsa-miR-125b-5p	1.23	Prevents aggrecan loss
hsa-miR-17-5p	0.82	Induces autophagy
hsa-miR-221-3p	0.57	Prevents ECM degradation
hsa-miR-92a-3p	0.48	Increases collagen deposition
hsa-miR-199a-3p	0.23	Anti-catabolic
**Total**	**35.94**	
*Destructive*		
hsa-miR-21-5p	0.88	Negatively regulates chondrogenesis
hsa-miR-16-5p	0.82	Cartilage degradation
hsa-miR-30b-5p	0.44	Pro-apoptotic, ECM degradation
hsa-miR-138-5p	0.34	Cartilage degradation
hsa-miR-483-5	0.26	Chondrocyte hypertrophy, ECM degradation and cartilage angiogenesis
hsa-miR-146a-5p	0.19	Activator in early OA
hsa-miR-34a-5p	0.17	Apoptosis
**Total**	**3.10**	
*Dual*		
hsa-miR-145-5p	1.25	Regulates chondrocyte proliferation and fibrosis
**SYNOVIUM**		
*Protective*		
hsa-miR-29a-3p	0.64	Anti-fibrotic effects
*Destructive*		
hsa-miR-34a-5p	0.17	Enhances synovial inflammation
*Dual*		
hsa-miR-146a-5p	0.19	Enhances/Suppresses synovial inflammation
**MACROPHAGE**		
*M1*		
hsa-miR-145-5p	1.25	Pro-M1
hsa-miR-125b-5p	1.23	Pro-M1
**Total**	**2.48**	
*M2*		
hsa-miR-24-3p	13.17	Pro M2, blocks M1
hsa-miR-222-3p	7.59	Pro M2
hsa-let-7b-5p	0.71	Pro M2
hsa-miR-146a-5p	0.19	Pro M2, blocks M1
hsa-miR-34a-5p	0.17	Pro M2
**Total**	**21.83**	
**T CELL**		
*Pro-Activation*		
hsa-miR-19b-3p	1.35	Reduces PTEN repressor
hsa-miR-214-3p	1.32	Reduces PTEN repressor
hsa-miR-21-5p	0.88	Reduces PTEN repressor
hsa-miR-106a-5p	0.83	Represses IL10
hsa-miR-17-5p	0.82	Reduces PTEN repressor and promotes IFNγ
hsa-let-7b-5p	0.71	Represses IL10
hsa-miR-221-3p	0.57	Downregulates PIK3R1
hsa-miR-132-3p	0.40	Downregulates PIK3R1
**Total**	**6.88**	
*Anti-activation*		
hsa-miR-24-3p	13.17	Represses IFNγ in activated CD4+ and CD8+
hsa-miR-125b-5p	1.23	Maintains T cell naïve state
hsa-miR-342-3p	0.90	Downregulated upon activation
hsa-miR-146a-5p	0.19	Represses activation markers
**Total**	**15.48**	
*Dual*		
hsa-miR-31-5p	0.21	Upregulates IL2, downregulated with activation

## Data Availability

The raw data presented in this study are openly available in OSF at https://osf.io/62fdj/?view_only=bc28fe818dd5426eb31a2286f6253c48 (accessed on 24 June 2022).

## Supplementary Materials

The following supporting information can be downloaded at: https://www.mdpi.com/article/10.3390/biology11111632/s1, Table S1: Pooled SF factors; Table S2: SF-treated BMSCs secreted factors; Table S3: GO terms defined by SF-treated BMSCs secreted factors; Table S4: miRNAs detected in SF-treated BMSC-EVs; Table S5: First quartile BMSCs EV-miRNAs targets; Table S6: Univocal miRNA-mRNA targets.
