# Supplementary material for: Molecular Characterization of Secreted Factors and Extracellular Vesicles-Embedded miRNAs from Bone Marrow-Derived Mesenchymal Stromal Cells in Presence of Synovial Fluid from Osteoarthritis Patients
Source: Biology (Basel). 2022 Nov 8;11(11):1632. doi: 10.3390/biology11111632 (PMC9687557; doi:10.3390/biology11111632)
Supplement: Supplementary file 1 [file biology-11-01632-s001.zip › Supplementary Table S1.pdf]

Supplementary Table S1 – Pooled SF factors

TYPE	FACTOR	(pg/ml)	
GF	IGFBP4	248,050	Insulin-like growth factor-binding protein 4
REC	VCAM1	109,797	Vascular cell adhesion protein 1
CYT	ICAM2	109,414	Intercellular adhesion molecule 2
GF	IGFBP2	44,358	Insulin-like growth factor-binding protein 2
CYT	PLG	42,376	Plasminogen
GF	IGFBP3	42,150	Insulin-like growth factor-binding protein 3
CYT	SIGLEC5	32,371	Sialic acid-binding Ig-like lectin 5
GF	TGFB1	29,154	Transforming growth factor beta-1
INF	TIMP2	25,220	Metalloproteinase inhibitor 2
REC	SELL	20,525	L-selectin
INF	IL6R	19,933	Interleukin-6 receptor subunit alpha
GF	BMP4	19,550	Bone morphogenetic protein 4
INF	TNFRSF1A	19,497	Tumor necrosis factor receptor superfamily member 1A
GF	CSF1R	19,117	Macrophage colony-stimulating factor 1 receptor
CYT	IL6ST	15,002	Interleukin-6 receptor subunit beta
CHE	IFNL1	13,977	Interferon lambda-1
REC	TNFRSF17	12,355	Tumor necrosis factor receptor superfamily member 17
REC	PLAUR	11,597	Urokinase plasminogen activator surface receptor
INF	TNFRSF1B	10,977	Tumor necrosis factor receptor superfamily member 1B
INF	TIMP1	9,879	Metalloproteinase inhibitor 1
CYT	CTSS	9,582	Cathepsin S
INF	ICAM1	9,291	Intercellular adhesion molecule 1
CYT	PARN	9,230	Poly(A)-specific ribonuclease PARN
CYT	SERPINE1	7,097	Plasminogen activator inhibitor 1
GF	IGFBP6	6,732	Insulin-like growth factor-binding protein 6
INF	CCL5	6,700	C-C motif chemokine 5
REC	CD14	5,872	Monocyte differentiation antigen CD14
GF	IGFBP1	5,671	Insulin-like growth factor-binding protein 1
REC	PI3	5,319	Elafin
GF	VEGFA	4,862	Vascular endothelial growth factor A
REC	ALCAM	4,815	CD166 antigen
GF	KDR	4,809	Vascular endothelial growth factor receptor 2
INF	PDGFB	4,537	Platelet-derived growth factor subunit B
CYT	FLT1	4,535	Vascular endothelial growth factor receptor 1
CYT	INHBA	4,386	Inhibin beta A chain
GF	FGF4	4,059	Fibroblast growth factor 4
GF	FGF7	3,499	Fibroblast growth factor 7
GF	KIT	3,386	Mast/stem cell growth factor receptor Kit
REC	PDGFRB	3,182	Platelet-derived growth factor receptor beta
REC	LCN2	3,153	Neutrophil gelatinase-associated lipocalin
CHE	MIF	3,061	Macrophage migration inhibitory factor
CYT	ANG	2,814	Angiogenin
INF	IL11	2,617	Interleukin-11
CHE	PF4	2,361	Platelet factor 4
GF	NTF3	2,350	Neurotrophin-3

CHE	XCL1	2,350	Lymphotactin
GF	BMP7	2,249	Bone morphogenetic protein 7
CYT	FCGR2B	2,221	Low affinity immunoglobulin gamma Fc region receptor II-b
REC	TNFRSF14	2,201	Tumor necrosis factor receptor superfamily member 14
CHE	CXCL16	2,179	C-X-C motif chemokine 16
GF	FGF2	1,931	Fibroblast growth factor 2
REC	TNFRSF21	1,902	Tumor necrosis factor receptor superfamily member 21
REC	PECAM1	1,877	Platelet endothelial cell adhesion molecule
CHE	CCL27	1,820	C-C motif chemokine 27
CYT	IL13RA2	1,736	Interleukin-13 receptor subunit alpha-2
CHE	MST1	1,651	Hepatocyte growth factor-like protein
CYT	IL13RA1	1,625	Interleukin-13 receptor subunit alpha-1
REC	LYVE1	1,494	Lymphatic vessel endothelial hyaluronic acid receptor 1
GF	EGFR	1,484	Epidermal growth factor receptor
REC	SELE	1,367	E-selectin
CYT	RETN	1,353	Resistin
REC	IL21R	1,248	Interleukin-21 receptor
GF	INS	1,234	Insulin
CYT	TREM1	1,186	Triggering receptor expressed on myeloid cells 1
INF	CCL2	1,100	C-C motif chemokine 2
CYT	CDH1	1,075	Cadherin-1
CHE	CXCL11	1,068	C-X-C motif chemokine 11
CYT	IL23A	1,060	Interleukin-23 subunit alpha
REC	TNFRSF10C	993	Tumor necrosis factor receptor superfamily member 10C
GF	BMP5	969	Bone morphogenetic protein 5
GF	HGF	962	Hepatocyte growth factor
GF	AREG	909	Amphiregulin
CYT	IL2RB	904	Interleukin-2 receptor subunit beta
CYT	CED	876	Diaphyseal Dysplasia 1
REC	ERBB3	812	Receptor tyrosine-protein kinase erbB-3
GF	NGFR	811	Tumor necrosis factor receptor superfamily member 16
REC	CNTN2	811	Contactin-2
REC	CEACAM1	809	Carcinoembryonic antigen-related cell adhesion molecule 1
REC	MOK	792	MAPK/MAK/MRK overlapping kinase
REC	ENG	777	Endoglin
CHE	CCL26	761	C-C motif chemokine 26
REC	FAS	753	Tumor necrosis factor receptor superfamily member 6
CHE	LIF	710	Leukemia inhibitory factor
CHE	CCL21	691	C-C motif chemokine 21
GF	GDF15	669	Growth/differentiation factor 15
GF	TGFB3	632	Transforming growth factor beta-3
GF	KITLG	606	Kit ligand
CYT	LGALS7	601	Galectin-7
GF	IGF1	600	Insulin-like growth factor I
CYT	TEK	588	Angiopoietin-1 receptor
REC	CD80	588	T-lymphocyte activation antigen CD80
INF	CCL15	558	C-C motif chemokine 15

CHE	SPP1	549	Osteopontin
GF	NTF4	525	Neurotrophin-4
REC	SCARB2	516	Lysosome membrane protein 2
GF	GDNF	471	Glial cell line-derived neurotrophic factor
INF	IL1RN	459	Interleukin-1 receptor antagonist protein
GF	TGFA	444	Protransforming growth factor alpha
REC	NRG1	425	Pro-neuregulin-1, membrane-bound isoform
INF	CXCL9	410	C-X-C motif chemokine 9
REC	IL17RA	408	Interleukin-17 receptor A
REC	TNFRSF18	404	Tumor necrosis factor receptor superfamily member 18
GF	PGF	391	Placenta growth factor
INF	CSF2	386	Granulocyte-macrophage colony-stimulating factor
GF	PDGFA	384	Platelet-derived growth factor subunit A
REC	MICA	349	MHC class I polypeptide-related sequence A
INF	IL16	339	Pro-interleukin-16
CHE	CCL28	336	C-C motif chemokine 28
REC	TYRO3	323	Tyrosine-protein kinase receptor TYRO3
CYT	DKK1	319	Dickkopf-related protein 1
CYT	PDGF1	318	Platelet-Derived Growth Factor A Chain
CYT	TNFRSF10D	313	Tumor necrosis factor receptor superfamily member 10D
CYT	NRCAM	310	Neuronal cell adhesion molecule
GF	FIGF	309	Vascular endothelial growth factor D
CYT	TGFB2	308	Transforming growth factor beta-2
CHE	TNFSF14	298	Tumor necrosis factor ligand superfamily member 14
CHE	IFNL2	274	Interferon lambda-2
REC	TNFRSF9	263	Tumor necrosis factor receptor superfamily member 9
CHE	IL18BP	242	Interleukin-18-binding protein
REC	TNFRSF8	228	Tumor necrosis factor receptor superfamily member 8
CHE	CXCL10	227	C-X-C motif chemokine 10
REC	FLT3LG	225	Fms-related tyrosine kinase 3 ligand
REC	CD40LG	225	CD40 ligand
INF	IL6	209	Interleukin-6
CHE	IL31	201	Interleukin-31
CYT	ANGPT1	200	Angiopoietin-1
CHE	CCL25	190	C-C motif chemokine 25
CHE	CXCL12	184	C-X-C motif chemokine 12
CYT	IL1RL1	182	Interleukin-1 receptor-like 1
GF	FLT4	179	Vascular endothelial growth factor receptor 3
CYT	IL17B	178	Interleukin-17B
INF	CCL24	171	C-C motif chemokine 24
CYT	SDF1	165	Stromal cell-derived factor 1
INF	CCL3	163	C-C motif chemokine 3
GF	PROK1	150	Prokineticin-1
CYT	VEGFC	134	Vascular endothelial growth factor C
REC	MICB	134	MHC class I polypeptide-related sequence B
CHE	AXL	131	Tyrosine-protein kinase receptor UFO
INF	IL2	122	Interleukin-2

REC	HAVCR1	120	Hepatitis A virus cellular receptor 1
CYT	CD40	114	Tumor necrosis factor receptor superfamily member 5
INF	IL1A	112	Interleukin-1 alpha
CYT	TDGF1	108	Teratocarcinoma-derived growth factor 1
GF	TNFRSF11B	104	Tumor necrosis factor receptor superfamily member 11B
INF	IL7	104	Interleukin-7
CYT	FST	103	Follistatin
CHE	CCL7	101	C-C motif chemokine 7
GF	NGF	99	Beta-nerve growth factor
INF	IL1B	99	Interleukin-1 beta
GF	GH1	94	Somatotropin
INF	IL12B	92	Interleukin-12 subunit beta
CYT	THPO	91	Thrombopoietin
INF	IFNG	86	Interferon gamma
INF	CCL11	85	Eotaxin
REC	IL10RB	74	Interleukin-10 receptor subunit beta
REC	EDA2R	70	Tumor necrosis factor receptor superfamily member 27
CYT	IL2RA	69	Interleukin-2 receptor subunit alpha
CHE	BTC	63	Probetacellulin
REC	ICAM3	60	Intercellular adhesion molecule 3
GF	HBEGF	57	Proheparin-binding EGF-like growth factor
INF	CCL4	57	C-C motif chemokine 4
INF	CXCL8	56	Interleukin-8
INF	CCL1	47	C-C motif chemokine 1
INF	LTA	46	Lymphotoxin-alpha
CHE	CCL8	41	C-C motif chemokine 8
CYT	EPCAM	38	Epithelial cell adhesion molecule
CYT	AGRP	33	Agouti-related protein
INF	IL4	32	Interleukin-4
GF	BDNF	31	Brain-derived neurotrophic factor
INF	IL10	30	Interleukin-10
INF	CXCL13	27	C-X-C motif chemokine 13
INF	IL15	24	Interleukin-15
CHE	CCL20	17	C-C motif chemokine 20
INF	IL5	16	Interleukin-5
INF	IL17A	11	Interleukin-17A
INF	IL13	9	Interleukin-13
REC	IL1R1	9	Interleukin-1 receptor type 1
CHE	IL17F	9	Interleukin-17F
CHE	CCL13	7	C-C motif chemokine 13
INF	CSF3	7	Granulocyte colony-stimulating factor
CHE	PPBP	5	Platelet basic protein
CYT	SHH	5	Sonic hedgehog protein
CHE	CCL23	4	Myeloid progenitor inhibitory factor
CHE	CCL16	4	C-C motif chemokine 16
CHE	CCL17	4	C-C motif chemokine 17
GF	EGF	3	Pro-epidermal growth factor

INF	CSF1	3	Macrophage colony-stimulating factor 1
INF	IL12A	2	Interleukin-12 subunit alpha
CYT	FASLG	1	Tumor necrosis factor ligand superfamily member 6
CHE	CCL22	1	C-C motif chemokine 22

CHE: Chemokine; CYT: Cytokine; GF: Growth factor; INF: Inflammation; REC: Receptor
