# Supplementary material for: Molecular Characterization of Secreted Factors and Extracellular Vesicles-Embedded miRNAs from Bone Marrow-Derived Mesenchymal Stromal Cells in Presence of Synovial Fluid from Osteoarthritis Patients
Source: Biology (Basel). 2022 Nov 8;11(11):1632. doi: 10.3390/biology11111632 (PMC9687557; doi:10.3390/biology11111632)
Supplement: Supplementary file 1 [file biology-11-01632-s001.zip › Supplementary Table S2.pdf]

Supplementary Table S2 – SF-treated BMSCs secreted factors

		pg/milione cellule					
TYPE	FACTOR	B1	B2	B3	MEAN	SD	FUNCTION
GF	IGFBP4	109,869	131,569	124,587	122,009	9,045	Insulin-like growth factor-binding protein 4
GF	IGFBP3	84,091	117,198	102,025	101,105	13,532	Insulin-like growth factor-binding protein 3
GF	TGFB1	39,339	32,615	30,189	34,048	3,870	Transforming growth factor beta-1
INF	TIMP2	22,096	28,331	30,462	26,963	3,550	Metalloproteinase inhibitor 2
CHE	IFNL1	19,399	20,613	22,381	20,798	1,225	Interferon lambda-1
CHE	IL9	1,770	24,217	16,270	14,086	9,293	Interleukin-9
INF	TIMP1	12,116	14,563	14,503	13,727	1,140	Metalloproteinase inhibitor 1
GF	BMP4	7,872	10,892	20,075	12,946	5,190	Bone morphogenetic protein 4
CYT	SERPINE1	10,110	14,584	13,253	12,649	1,876	Plasminogen activator inhibitor 1
GF	IGFBP2	6,439	14,130	10,661	10,410	3,145	Insulin-like growth factor-binding protein 2
GF	VEGFA	7,794	12,442	10,732	10,323	1,919	Vascular endothelial growth factor A
CHE	PF4	9,652	10,111	8,745	9,503	567	Platelet factor 4
GF	IGFBP6	6,466	10,314	9,509	8,763	1,657	Insulin-like growth factor-binding protein 6
CHE	MIF	3,604	4,526	4,973	4,368	570	Macrophage migration inhibitory factor
REC	VCAM1	4,131	5,719	2,852	4,234	1,173	Vascular cell adhesion protein 1
CYT	INHBA	4,344	4,350	3,770	4,155	272	Inhibin beta A chain
CHE	XCL1	2,921	4,047	5,177	4,048	921	Lymphotactin
CHE	CCL27	4,283	2,370	3,979	3,544	840	C-C motif chemokine 27
CHE	CXCL16	2,374	3,787	3,932	3,364	702	C-X-C motif chemokine 16
INF	TNFRSF1A	2,640	3,403	3,410	3,151	361	Tumor necrosis factor receptor superfamily member 1A
CHE	MST1	3,356	1,822	2,150	2,443	659	Hepatocyte growth factor-like protein
CHE	CCL26	2,212	932	4,108	2,417	1,305	C-C motif chemokine 26
CHE	CCL21	2,552	3,078	1,594	2,408	614	C-C motif chemokine 21
CHE	CXCL11	2,165	3,168	1,466	2,266	698	C-X-C motif chemokine 11
REC	PLAUR	1,515	2,482	1,641	1,879	429	Urokinase plasminogen activator surface receptor
CYT	ANGPT1	1,553	2,005	1,837	1,798	187	Angiopoietin-1
GF	BMP7	2,239	327	1,766	1,444	813	Bone morphogenetic protein 7
CYT	IL6ST	1,239	1,842	1,090	1,390	325	Interleukin-6 receptor subunit beta
GF	HGF	1,058	1,462	1,443	1,321	186	Hepatocyte growth factor
GF	FGF4	1,536	1,484	729	1,250	369	Fibroblast growth factor 4
CYT	ANG	971	1,427	1,301	1,233	193	Angiogenin
REC	ALCAM	956	1,209	1,239	1,135	127	CD166 antigen
CYT	FST	690	1,260	1,284	1,078	274	Follistatin
CYT	CTSS	808	1,256	1,150	1,071	191	Cathepsin S
GF	KDR	1,078	887	1,102	1,022	96	Vascular endothelial growth factor receptor 2
CHE	TNFSF14	1,027	1,012	846	962	82	Tumor necrosis factor ligand superfamily member 14
CHE	SPP1	906	1,019	861	929	67	Osteopontin
CHE	CCL25	573	995	1,075	881	220	C-C motif chemokine 25
CYT	FLT1	1,038	816	725	860	131	Vascular endothelial growth factor receptor 1
INF	CCL2	662	814	1,053	843	161	C-C motif chemokine 2
CYT	CDH1	599	1,073	680	784	207	Cadherin-1
GF	FGF7	726	1,138	482	782	271	Fibroblast growth factor 7
GF	IGFBP1	457	922	873	750	209	Insulin-like growth factor-binding protein 1
CHE	LIF	665	1,170	337	724	343	Leukemia inhibitory factor

CYT	CED	544	794	699	679	103	Diaphyseal Dysplasia 1
CYT	IL13RA2	171	1,001	846	673	361	Interleukin-13 receptor subunit alpha-2
INF	LTA	1,346	248	202	599	529	Lymphotoxin-alpha
CYT	IL23A	446	622	629	565	84	Interleukin-23 subunit alpha
GF	BMP5	415	607	580	534	85	Bone morphogenetic protein 5
CHE	IFNL2	445	467	645	519	90	Interferon lambda-2
INF	IL6	430	515	609	518	73	Interleukin-6
CHE	CXCL10	397	387	600	461	98	C-X-C motif chemokine 10
GF	INS	468	725	173	455	226	Insulin
GF	GDF15	351	486	350	396	64	Growth/differentiation factor 15
CHE	AXL	468	122	562	384	189	Tyrosine-protein kinase receptor UFO
CHE	BTC	337	406	404	383	32	Probetacellulin
GF	NTF4	317	353	383	351	27	Neurotrophin-4
CYT	IL17B	317	474	127	306	142	Interleukin-17B
CHE	IL18BP	256	520	125	300	164	Interleukin-18-binding protein
CHE	CXCL12	232	339	310	294	45	C-X-C motif chemokine 12
CYT	SDF1	225	343	312	293	50	Stromal cell-derived factor 1
INF	IL1RN	224	286	329	280	43	Interleukin-1 receptor antagonist protein
INF	TNFRSF1B	211	312	313	279	48	Tumor necrosis factor receptor superfamily member 1B
GF	NTF3	265	152	351	256	81	Neurotrophin-3
INF	IL6R	202	264	220	229	26	Interleukin-6 receptor subunit alpha
GF	AREG	231	187	250	223	26	Amphiregulin
GF	KIT	167	237	239	214	33	Mast/stem cell growth factor receptor Kit
GF	GDNF	259	85	294	213	91	Glial cell line-derived neurotrophic factor
REC	CD14	175	231	216	207	24	Monocyte differentiation antigen CD14
INF	CCL5	149	242	213	202	39	C-C motif chemokine 5
GF	NGFR	272	142	159	191	58	Tumor necrosis factor receptor superfamily member 16
INF	ICAM1	146	195	193	178	22	Intercellular adhesion molecule 1
INF	IL1A	171	160	198	177	16	Interleukin-1 alpha
GF	IGF1	231	116	140	162	50	Insulin-like growth factor I
GF	EGFR	113	185	160	153	30	Epidermal growth factor receptor
INF	CXCL8	136	109	180	142	29	Interleukin-8
INF	IL16	112	207	76	132	55	Pro-interleukin-16
GF	FIGF	52	151	161	122	49	Vascular endothelial growth factor D
CHE	IL31	57	286	14	119	119	Interleukin-31
INF	CXCL9	102	109	144	118	18	C-X-C motif chemokine 9
CYT	VEGFC	110	194	39	114	63	Vascular endothelial growth factor C
CYT	DKK1	97	199	39	112	66	Dickkopf-related protein 1
GF	KITLG	98	121	95	105	12	Kit ligand
GF	TNFRSF11B	101	86	89	92	7	Tumor necrosis factor receptor superfamily member 11B
CHE	CCL7	110	57	89	85	22	C-C motif chemokine 7
CHE	CCL8	41	114	98	85	31	C-C motif chemokine 8
GF	TGFB3	83	36	117	79	33	Transforming growth factor beta-3
INF	IL15	73	48	94	72	19	Interleukin-15
INF	IL7	85	37	81	67	22	Interleukin-7
INF	CCL1	5	137	36	59	56	C-C motif chemokine 1
GF	TGFA	40	25	103	56	34	Protransforming growth factor alpha

REC	FAS	39	45	53	46	6	Tumor necrosis factor receptor superfamily member 6
CHE	CCL18	37	52	48	46	6	C-C motif chemokine 18
CHE	CCL20	48	40	48	45	4	C-C motif chemokine 20
GF	PROK1	39	40	54	44	7	Prokineticin-1
GF	PGF	36	54	39	43	8	Placenta growth factor
INF	IL2	25	27	65	39	19	Interleukin-2
GF	FLT4	25	36	52	37	11	Vascular endothelial growth factor receptor 3
CYT	EPCAM	31	31	43	35	5	Epithelial cell adhesion molecule
INF	CSF2	35	37	24	32	6	Granulocyte-macrophage colony-stimulating factor
INF	CSF1	34	26	36	32	4	Macrophage colony-stimulating factor 1
CHE	CCL14	20	24	40	28	9	C-C motif chemokine 14
INF	IL4	16	18	36	23	9	Interleukin-4
INF	IFNG	17	29	22	23	5	Interferon gamma
CHE	CCL13	18	22	27	22	4	C-C motif chemokine 13
INF	CSF3	23	25	14	21	5	Granulocyte colony-stimulating factor
REC	ENG	4	30	23	19	11	Endoglin
GF	GH1	11	31	12	18	9	Somatotropin
CYT	IL2RA	14	22	17	18	4	Interleukin-2 receptor subunit alpha
GF	PDGFA	24	4	23	17	9	Platelet-derived growth factor subunit A
INF	CCL11	26	9	12	15	8	Eotaxin
CHE	CCL17	15	19	10	15	4	C-C motif chemokine 17
CHE	CXCL5	12	17	8	12	4	C-X-C motif chemokine 5
GF	BDNF	10	10	14	11	2	Brain-derived neurotrophic factor
CYT	SHH	13	4	13	10	4	Sonic hedgehog protein
CHE	CCL16	7	6	17	10	5	C-C motif chemokine 16
CHE	PPBP	9	11	8	9	1	Platelet basic protein
REC	TNFRSF21	11	7	7	8	2	Tumor necrosis factor receptor superfamily member 21
INF	CCL24	5	17	2	8	6	C-C motif chemokine 24
INF	IL1B	4	9	7	7	2	Interleukin-1 beta
INF	CCL4	3	5	2	3	1	C-C motif chemokine 4
GF	NGF	1	5	4	3	2	Beta-nerve growth factor
INF	CXCL13	3	4	2	3	1	C-X-C motif chemokine 13
GF	EGF	1	1	1	1	0	Pro-epidermal growth factor
INF	IL12A	1	1	1	1	0	Interleukin-12 subunit alpha

CHE: Chemokine; CYT: Cytokine; GF: Growth factor; INF: Inflammation; REC: Receptor
