# Supplementary material for: Molecular Characterization of Secreted Factors and Extracellular Vesicles-Embedded miRNAs from Bone Marrow-Derived Mesenchymal Stromal Cells in Presence of Synovial Fluid from Osteoarthritis Patients
Source: Biology (Basel). 2022 Nov 8;11(11):1632. doi: 10.3390/biology11111632 (PMC9687557; doi:10.3390/biology11111632)
Supplement: Supplementary file 1 [file biology-11-01632-s001.zip › Supplementary Table S3.pdf]

Supplementary Table S3 – GO terms defined by SF-treated BMSCs secreted factors

- GO:0040011 Locomotion (found) 65 (database) 1251 (FDR) 3.26e-41
NGFR,MIF,CCL17,TGFB1,HGF,CCL2,CCL1,CCL13,KITLG,BMP4,CCL21,CCL27,IL1B,EPCAM,KDR,ICAM1,FGF7,EGFR,FLT1,CXCL13,KIT,CXCL16,VCAM1,CXCL5,PPBP,PF4,SHH,FIGF,AXL,IGFBP6,CCL11,IL16,IL12A,CXCL10,ALCAM,CXCL8,CXCL11,ANG,PLAUR,PDGFA,CCL20,CXCL9,XCL1,IL6R,ENG,CCL7,CCL25,CCL8,CCL26,CXCL12,BMP7,IL6,NTF3,CCL24,GDNF,BDNF,ANGPT1,PGF,CCL5,CCL16,VEGFA,CCL14,CCL18,VEGFC,CCL4

- GO:0006935 Chemotaxis 54 545 1.95e-46
NGFR,MIF,CCL17,HGF,CCL2,CCL1,CCL13,BMP4,CCL21,CCL27,IL1B,FGF7,FLT1,CXCL13,KIT,CXCL16,CXCL5,PPBP,PF4,SHH,FIGF,CCL11,IL16,CXCL10,ALCAM,CXCL8,CXCL11,PLAUR,PDGFA,CCL20,CXCL9,XCL1,IL6R,ENG,CCL7,CCL25,CCL8,CCL26,CXCL12,BMP7,IL6,NTF3,CCL24,GDNF,BDNF,ANGPT1,PGF,CCL5,CCL16,VEGFA,CCL14,CCL18,VEGFC,CCL4

- GO:0071621 Granulocyte chemotaxis 28 81 6.81e-36
CCL17,CCL2,CCL1,CCL13,CCL21,IL1B,CXCL13,CXCL5,PPBP,PF4,CCL11,CXCL10,CXCL8,CXCL11,CCL20,CXCL9,XCL1,CCL7,CCL25,CCL8,CCL26,CCL24,CCL5,CCL16,VEGFA,CCL14,CCL18,CCL4

- GO:0048247 Lymphocyte chemotaxis 23 50 1.40e-31
CCL17,CCL2,CCL1,CCL13,CCL21,CXCL13,CXCL16,CCL11,CXCL10,CXCL11,CCL20,XCL1,CCL7,CCL25,CCL8,CCL26,CXCL12,CCL24,CCL5,CCL16,CCL14,CCL18,CCL4

- GO:0002548 Monocyte chemotaxis 21 43 3.61e-29
CCL17,CCL2,CCL1,CCL13,CCL21,FLT1,CCL11,CCL20,XCL1,IL6R,CCL7,CCL25,CCL8,CCL26,IL6,CCL24,CCL5,CCL16,CCL14,CCL18,CCL4

- GO:0071363 Cellular response to growth factor stimulus 30 494 7.74e-19
FGF4,NGFR,TGFB1,HGF,CCL2,TGFB3,BMP4,GDF15,FST,FLT4,KDR,FGF7,EGFR,FLT1,CXCL13,FIGF,CXCL8,XCL1,NGF,BMP5,ENG,TNFRSF1B,BMP7,NTF3,BDNF,PGF,NTF4,CCL5,VEGFA,VEGFC

- GO:0070102 interleukin-6-mediated signaling pathway 3 16 0.0029

IL6R,IL6ST,IL6

- GO:0043567 Regulation of insulin-like growth factor receptor signaling pathway 8 23 5.50e-10

IGFBP2,IGFBP4,IGFBP1,IGFBP6,IGF1,GH1,BMP5,IGFBP3

- GO:0030198 Extracellular matrix organization 13 338 7.90e-06
TIMP1,TGFB1,SERPINE1,CDH1,TIMP2,KDR,ICAM1,VCAM1,TNFRSF11B,PDGFA,CTSS,ENG,SPP1
