# Supplementary material for: Molecular Characterization of Secreted Factors and Extracellular Vesicles-Embedded miRNAs from Bone Marrow-Derived Mesenchymal Stromal Cells in Presence of Synovial Fluid from Osteoarthritis Patients
Source: Biology (Basel). 2022 Nov 8;11(11):1632. doi: 10.3390/biology11111632 (PMC9687557; doi:10.3390/biology11111632)
Supplement: Supplementary file 1 [file biology-11-01632-s001.zip › Supplementary Table S4.pdf]

Supplementary Table S4 – miRNAs detected in SF-treated BMSC-EVs

miRBase ID	CRT					Weight %
	B1	B2	B3	Mean	SD	
**hsa-miR-518f-3p**	**9.26**	**12.46**	**13.80**	**11.84**	**1.90**	**17.75728**
**hsa-miR-24-3p**	**12.47**	**12.14**	**12.20**	**12.27**	**0.15**	**13.16838**
**hsa-miR-193b-3p**	**13.04**	**12.78**	**12.95**	**12.92**	**0.11**	**8.388070**
**hsa-miR-222-3p**	**13.26**	**13.12**	**12.81**	**13.06**	**0.19**	**7.591257**
**hsa-miR-1274B**	**13.12**	**13.18**	**13.33**	**13.21**	**0.09**	**6.863791**
**hsa-miR-574-3p**	**13.50**	**13.24**	**13.55**	**13.43**	**0.14**	**5.882132**
**hsa-miR-191-5p**	**13.43**	**13.53**	**13.56**	**13.51**	**0.06**	**5.582864**
**hsa-miR-484**	**14.17**	**13.74**	**13.90**	**13.94**	**0.18**	**4.142033**
**hsa-miR-320a-3p**	**14.34**	**13.94**	**14.30**	**14.19**	**0.18**	**3.468564**
**hsa-miR-197-3p**	**15.20**	**14.51**	**14.89**	**14.86**	**0.28**	**2.181023**
**hsa-miR-19b-3p**	**15.44**	**15.55**	**15.68**	**15.55**	**0.10**	**1.352228**
**hsa-miR-214-3p**	**15.47**	**15.49**	**15.81**	**15.59**	**0.16**	**1.317989**
**hsa-miR-99a-5p**	**15.69**	**15.67**	**15.63**	**15.66**	**0.03**	**1.254117**
**hsa-miR-145-5p**	**15.61**	**15.59**	**15.80**	**15.67**	**0.09**	**1.250356**
**hsa-miR-125b-5p**	**15.76**	**15.67**	**15.65**	**15.69**	**0.05**	**1.226040**
**hsa-miR-1274A**	**15.80**	**15.93**	**16.14**	**15.96**	**0.14**	**1.021016**
**hsa-miR-627-5p**	**17.05**	**14.73**	**16.22**	**16.00**	**0.96**	**0.992408**
**hsa-miR-342-3p**	**16.22**	**15.80**	**16.38**	**16.13**	**0.25**	**0.904174**
**hsa-miR-409-3p**	**16.19**	**16.33**	**15.94**	**16.15**	**0.16**	**0.891932**
**hsa-miR-21-5p**	**15.80**	**16.24**	**16.46**	**16.17**	**0.28**	**0.884135**
**hsa-miR-106a-5p**	**16.02**	**16.42**	**16.33**	**16.25**	**0.17**	**0.832009**
**hsa-miR-16-5p**	**16.37**	**16.29**	**16.19**	**16.28**	**0.07**	**0.816961**
**hsa-miR-17-5p**	**16.10**	**16.36**	**16.39**	**16.28**	**0.13**	**0.815264**
**hsa-let-7b-5p**	**16.39**	**16.40**	**16.66**	**16.48**	**0.12**	**0.710056**
**hsa-miR-29a-3p**	**16.71**	**16.83**	**16.39**	**16.64**	**0.19**	**0.635518**
**hsa-miR-30c-5p**	**16.48**	**16.81**	**16.81**	**16.70**	**0.16**	**0.610052**
**hsa-miR-221-3p**	**16.79**	**16.75**	**16.86**	**16.80**	**0.05**	**0.569594**
**hsa-miR-92a-3p**	**17.12**	**16.93**	**17.10**	**17.05**	**0.08**	**0.478969**
**hsa-miR-30b-5p**	**16.93**	**17.20**	**17.35**	**17.16**	**0.17**	**0.444629**
**hsa-miR-20a-5p**	**16.92**	**17.28**	**17.43**	**17.21**	**0.21**	**0.428492**
**hsa-miR-132-3p**	**17.31**	**17.13**	**17.47**	**17.30**	**0.14**	**0.402206**
**hsa-miR-618**	**13.41**	**14.78**	**24.32**	**17.50**	**4.85**	**0.350464**
**hsa-miR-138-5p**	**17.39**	**17.43**	**17.87**	**17.56**	**0.22**	**0.335722**
**hsa-miR-382-5p**	**18.55**	**17.53**	**17.41**	**17.83**	**0.51**	**0.279323**
**hsa-miR-663b**	**17.74**	**17.68**	**18.19**	**17.87**	**0.23**	**0.271308**
**hsa-miR-483-5**	**18.29**	**17.46**	**17.99**	**17.91**	**0.34**	**0.264011**
**hsa-miR-199a-3p**	**18.14**	**18.30**	**17.86**	**18.10**	**0.18**	**0.231327**
**hsa-miR-520e-3p**	**15.88**	**17.10**	**21.67**	**18.22**	**2.49**	**0.213307**
**hsa-miR-31-5p**	**18.39**	**18.44**	**17.85**	**18.23**	**0.27**	**0.211883**
**hsa-miR-28-3p**	**18.39**	**18.51**	**18.18**	**18.36**	**0.14**	**0.193402**
**hsa-miR-146a-5p**	**18.47**	**18.77**	**18.02**	**18.42**	**0.31**	**0.185395**
**hsa-miR-720**	**18.69**	**18.31**	**18.31**	**18.44**	**0.18**	**0.183435**
**hsa-miR-193a-5p**	**18.27**	**18.37**	**18.71**	**18.45**	**0.19**	**0.181957**
**hsa-miR-34a-5p**	**19.15**	**18.58**	**17.84**	**18.52**	**0.54**	**0.172740**
**hsa-miR-376a-3p**	**18.51**	**18.59**	**18.58**	**18.56**	**0.03**	**0.168094**
**hsa-miR-186-5p**	**19.01**	**18.74**	**18.40**	**18.72**	**0.25**	**0.151005**
hsa-miR-376c-3p	18.90	18.83	18.43	18.72	0.20	0.150901
hsa-miR-152-3p	18.80	18.77	19.01	18.86	0.11	0.136692
hsa-let-7e-5p	19.64	18.69	18.59	18.97	0.47	0.126219
hsa-miR-29c-3p	18.76	20.74	17.75	19.08	1.24	0.117359
hsa-miR-662	17.67	21.31	18.27	19.08	1.59	0.117251
hsa-miR-146b-5p	19.33	19.20	18.85	19.13	0.20	0.113650

hsa-miR-99b-5p	19.33	19.33	18.89	19.18	0.21	0.109273
hsa-miR-30a-3p	19.08	19.65	19.35	19.36	0.24	0.096478
hsa-miR-149-5p	19.45	19.56	19.39	19.47	0.07	0.089685
hsa-miR-636	18.85	17.30	22.31	19.48	2.09	0.088696
hsa-miR-205-5p	19.84	21.49	17.22	19.52	1.76	0.08675
hsa-miR-302a-3p	16.12	22.29	20.22	19.54	2.57	0.085102
hsa-miR-425-5p	18.90	19.82	19.93	19.55	0.46	0.084788
hsa-miR-143-3p	19.68	19.60	19.64	19.64	0.03	0.079752
hsa-miR-331-3p	19.87	19.78	19.34	19.67	0.23	0.078129
hsa-miR-328-3p	19.47	19.88	19.84	19.73	0.18	0.074825
hsa-miR-224-5p	19.74	19.80	19.69	19.74	0.04	0.074154
hsa-miR-130a-3p	19.89	19.60	19.81	19.76	0.12	0.072998
hsa-miR-218-5p	20.11	19.73	19.82	19.89	0.17	0.067126
hsa-miR-886-5p	19.99	19.98	19.85	19.94	0.07	0.064749
hsa-miR-155-5p	20.03	20.37	19.71	20.04	0.27	0.060427
hsa-miR-1290	20.05	20.67	19.52	20.08	0.47	0.058775
hsa-miR-106b-5p	20.00	19.83	20.61	20.15	0.34	0.055991
hsa-let-7a-5p	19.75	20.81	19.90	20.15	0.47	0.055746
hsa-miR-30e-3p	19.45	20.29	20.77	20.17	0.54	0.055182
hsa-miR-365a-3p	19.87	20.40	20.25	20.17	0.22	0.055004
hsa-miR-212-3p	20.18	20.18	20.42	20.26	0.11	0.051713
hsa-miR-335-5p	19.98	20.18	20.74	20.30	0.32	0.050299
hsa-miR-26a-5p	20.53	20.49	19.94	20.32	0.27	0.049733
hsa-miR-1304-5p	23.65	20.63	16.75	20.34	2.83	0.048867
hsa-miR-339-5p	20.46	19.78	20.84	20.36	0.44	0.048306
hsa-miR-532-5p	20.66	20.50	20.14	20.44	0.22	0.045859
hsa-miR-125a-5p	20.56	20.59	20.19	20.45	0.18	0.045426
hsa-miR-590-5p	20.97	20.34	20.49	20.60	0.27	0.040959
hsa-miR-31-3p	20.75	20.90	20.42	20.69	0.20	0.038393
hsa-miR-10b-3p	20.84	20.81	20.68	20.78	0.07	0.036214
hsa-miR-345-5p	20.78	20.83	20.79	20.80	0.02	0.035649
hsa-miR-210-3p	20.37	21.22	20.98	20.86	0.36	0.034197
hsa-miR-127-3p	20.92	21.03	20.64	20.86	0.16	0.034118
hsa-miR-134-5p	20.87	20.80	21.01	20.89	0.09	0.033354
hsa-miR-130b-3p	20.70	20.97	21.22	20.97	0.21	0.031774
hsa-miR-19a-3p	20.99	21.04	21.06	21.03	0.03	0.030417
hsa-miR-660-5p	21.33	20.84	21.36	21.18	0.24	0.027457
hsa-miR-301a-3p	20.94	20.91	22.00	21.28	0.51	0.025483
hsa-let-7g-5p	21.25	21.22	21.41	21.30	0.08	0.025260
hsa-miR-370-3p	21.45	20.89	21.77	21.37	0.36	0.024025
hsa-miR-27b-3p	21.41	21.31	21.43	21.38	0.05	0.023754
hsa-miR-93-5p	21.19	21.42	21.55	21.39	0.15	0.023667
hsa-miR-7-1-3p	21.31	21.64	21.36	21.44	0.15	0.022919
hsa-miR-140-5p	21.42	21.58	21.33	21.44	0.10	0.022839
hsa-miR-625-3p	20.92	21.83	21.59	21.45	0.39	0.022713
hsa-miR-551b-3p	26.46	17.16	20.79	21.47	3.83	0.022338
hsa-miR-27a-3p	21.29	21.49	21.79	21.52	0.21	0.021622
hsa-miR-374a-5p	21.43	22.11	21.18	21.57	0.39	0.020823
hsa-miR-494-3p	21.69	21.59	21.59	21.62	0.05	0.020123
hsa-miR-339-3p	21.44	21.31	22.20	21.65	0.39	0.019755
hsa-miR-423-5p	21.85	21.21	22.01	21.69	0.34	0.019223
hsa-miR-30a-5p	21.79	21.64	21.89	21.77	0.10	0.018161
hsa-miR-23a-3p	21.40	21.64	22.31	21.78	0.38	0.018065
hsa-miR-195-5p	21.93	21.87	21.78	21.86	0.06	0.017106
hsa-miR-15b-5p	22.06	21.86	21.68	21.87	0.15	0.017008
hsa-miR-323a-3p	21.98	21.92	21.92	21.94	0.03	0.016206

hsa-miR-193b-5p	21.90	21.85	22.25	22.00	0.18	0.015528
hsa-miR-25-3p	21.46	21.91	22.71	22.03	0.51	0.015229
hsa-miR-99b-3p	21.56	22.13	22.39	22.03	0.34	0.015205
hsa-miR-410-3p	21.70	22.58	21.85	22.05	0.38	0.015020
hsa-miR-432-3p	21.86	22.52	21.91	22.10	0.30	0.014478
hsa-miR-34b-3p	21.89	22.59	22.22	22.23	0.28	0.013191
hsa-miR-532-3p	22.42	22.39	21.88	22.23	0.25	0.013191
hsa-miR-26b-5p	22.45	22.55	21.74	22.25	0.36	0.013069
hsa-let-7c-5p	22.38	22.57	21.92	22.29	0.27	0.012694
hsa-miR-181a-5p	22.39	22.33	22.21	22.31	0.07	0.012528
hsa-miR-34a-3p	22.27	22.39	22.90	22.52	0.27	0.010789
hsa-miR-10a-5p	22.65	22.60	22.44	22.57	0.09	0.010467
hsa-miR-28-5p	23.38	22.55	21.82	22.58	0.64	0.010349
hsa-miR-664a-3p	22.94	22.77	22.26	22.66	0.29	0.009811
hsa-miR-361-5p	22.42	22.40	23.49	22.77	0.51	0.009118
hsa-miR-708-5p	23.53	22.95	22.04	22.84	0.61	0.008651
hsa-miR-215-5p	22.83	22.84	22.89	22.85	0.03	0.008579
hsa-miR-374b-5p	23.41	22.79	22.37	22.86	0.43	0.008557
hsa-miR-296-5p	23.10	22.39	23.20	22.89	0.36	0.008342
hsa-miR-766-3p	23.74	22.58	22.38	22.90	0.60	0.008323
hsa-miR-93-3p	22.46	23.26	23.18	22.97	0.36	0.007916
hsa-miR-454-3p	23.17	23.15	22.62	22.98	0.26	0.007851
hsa-miR-324-3p	22.64	23.01	23.33	22.99	0.28	0.007805
hsa-miR-192-5p	22.61	23.48	22.94	23.01	0.36	0.007696
hsa-miR-1260a	22.53	22.28	24.25	23.02	0.87	0.007659
hsa-miR-539-5p	23.62	22.92	22.88	23.14	0.34	0.007031
hsa-miR-148a-3p	23.45	23.66	22.91	23.34	0.32	0.006125
hsa-miR-330-3p	23.97	23.56	22.74	23.42	0.51	0.005786
hsa-miR-886-3p	22.73	24.79	22.82	23.45	0.95	0.005682
hsa-miR-629-3p	22.88	24.47	23.04	23.46	0.72	0.005630
hsa-miR-411-5p	23.83	24.50	22.74	23.69	0.72	0.004798
hsa-miR-194-5p	25.62	17.47	27.99	23.70	4.50	0.004789
hsa-miR-744-5p	24.53	23.34	23.46	23.78	0.53	0.004524
hsa-miR-487b-3p	23.45	23.71	24.22	23.79	0.32	0.004477
hsa-miR-223-3p	22.73	24.40	24.29	23.81	0.76	0.004436
hsa-miR-103a-3p	23.80	24.82	23.17	23.93	0.68	0.004079
hsa-miR-491-5p	24.39	23.98	23.48	23.95	0.37	0.004012
hsa-miR-485-3p	23.60	24.40	23.90	23.96	0.33	0.003973
hsa-miR-185-5p	24.40	23.74	23.88	24.01	0.29	0.003858
hsa-miR-1271-5p	23.31	25.22	23.63	24.05	0.84	0.003741
hsa-miR-493-3p	24.39	23.64	24.14	24.05	0.31	0.003734
hsa-miR-30d-3p	24.92	23.55	23.79	24.09	0.60	0.003651
hsa-miR-433-3p	23.70	24.36	24.25	24.10	0.29	0.003611
hsa-miR-455-5p	24.91	24.16	23.31	24.13	0.66	0.003552
hsa-miR-452-5p	23.76	25.21	23.79	24.25	0.68	0.003251
hsa-miR-615-3p	24.10	24.52	24.24	24.29	0.17	0.003172
hsa-miR-942-5p	23.83	25.50	23.76	24.36	0.80	0.003016
hsa-miR-133a-3p	24.72	24.89	23.81	24.47	0.48	0.002791
hsa-miR-140-3p	25.33	24.25	24.13	24.57	0.54	0.002611
hsa-miR-20b-5p	25.09	24.24	24.57	24.63	0.35	0.002500
hsa-miR-425-3p	25.14	25.39	24.43	24.99	0.41	0.001956
hsa-miR-214-5p	25.54	25.44	24.22	25.07	0.60	0.001850
hsa-miR-615-5p	24.45	24.88	25.92	25.08	0.62	0.001831
hsa-miR-203a-3p	25.20	24.37	26.14	25.24	0.72	0.001645
hsa-miR-1226-5p	24.54	25.43	25.77	25.25	0.52	0.001634
hsa-miR-10b-5p	26.01	26.04	23.95	25.33	0.98	0.001537

hsa-miR-889-3p	25.44	26.20	24.83	25.49	0.56	0.001380
hsa-miR-335-3p	25.87	25.20	25.51	25.53	0.27	0.001344
hsa-miR-628-3p	25.37	25.93	25.48	25.59	0.24	0.001287
hsa-miR-483-3p	27.78	24.95	24.50	25.74	1.45	0.001158
hsa-miR-589-3p	26.40	25.77	25.52	25.90	0.37	0.001042
hsa-miR-874-3p	25.06	25.90	26.75	25.90	0.69	0.001038
hsa-miR-222-5p	26.33	25.74	25.64	25.90	0.31	0.001036
hsa-miR-424-3p	27.39	24.73	26.10	26.07	1.08	0.000920
hsa-miR-1285-3p	26.08	26.35	26.27	26.23	0.11	0.000825
hsa-miR-136-3p	26.52	27.19	25.19	26.30	0.83	0.000788
hsa-miR-148b-3p	26.74	25.86	26.77	26.46	0.42	0.000707
hsa-miR-24-2-5p	27.84	25.87	25.71	26.47	0.97	0.000698
hsa-miR-126-3p	26.02	26.59	27.08	26.56	0.43	0.000656
hsa-miR-650	26.73	27.08	25.93	26.58	0.48	0.000649
hsa-miR-25-5p	26.44	27.78	26.52	26.91	0.61	0.000514
hsa-miR-372-3p	29.20	25.38	26.45	27.01	1.61	0.000481
hsa-miR-193a-3p	26.90	27.47	26.82	27.06	0.29	0.000464
hsa-miR-130b-5p	26.03	26.88	28.57	27.16	1.05	0.000434
hsa-miR-18a-5p	27.13	28.12	26.93	27.39	0.52	0.000369
hsa-miR-502-3p	28.56	26.38	29.24	28.06	1.22	0.000233
hsa-miR-151a-5p	28.00	28.56	28.17	28.25	0.23	0.000204
hsa-miR-1300	27.65	28.84	28.26	28.25	0.48	0.000204
hsa-miR-1255b-5p	29.02	28.91	27.68	28.54	0.61	0.000167
hsa-miR-505-5p	29.15	27.32	29.53	28.67	0.97	0.000153
hsa-miR-125a-3p	28.22	31.54	29.12	29.63	1.40	7.85E-05
hsa-miR-26a-1-3p	30.89	29.99	29.98	30.29	0.43	4.96E-05
hsa-miR-191-3p	30.12	33.22	32.38	31.91	1.31	1.62E-05
hsa-miR-145-3p	32.66	34.60	30.66	32.64	1.61	9.73E-06

In **bold** miRNAs falling in the first quartile of abundance.
