# Supplementary material for: Molecular Characterization of Secreted Factors and Extracellular Vesicles-Embedded miRNAs from Bone Marrow-Derived Mesenchymal Stromal Cells in Presence of Synovial Fluid from Osteoarthritis Patients
Source: Biology (Basel). 2022 Nov 8;11(11):1632. doi: 10.3390/biology11111632 (PMC9687557; doi:10.3390/biology11111632)
Supplement: Supplementary file 1 [file biology-11-01632-s001.zip › Supplementary Table S5.pdf]

Supplementary Table S5 - First quartile BMSCs EV-miRNAs targets

miRNA	hsa-miR-518f-3p	hsa-miR-24-3p	hsa-miR-193b-3p	hsa-miR-222-3p	hsa-miR-574-3p	hsa-miR-191-5p
Genetic weight %	17.76	13.17	8.39	7.59	5.88	5.58
Target		ABCB9	AKR1C2	ABCG2	CLTC	BASP1
		ACVR1B	CCND1	ADAM1A	CUL2	CCND2
		AGPAT2	ESR1	ARID1A	EGFR	CDK6
		ARHGAP19	ETS1	BBC3	EP300	CDK9
		ATG4A	KIT	BMF	RAC1	CEBPB
		AURKB	KRAS	CDKN1B	RXRA	CTDSP2
		BCAR1	MAX	CDKN1C	SMAD4	EGR1
		BCL2L11	MCL1	CERS2	TGFB1	IL1A
		BRCA1	MYB	CORO1A		LRRC8A
		CARD10	NF1	DICER1		MDM4
		CCNA2	PLAU	DIRAS3		NDST1
		CCND1	PRAP1	DKK2		NOTCH2
		CDK1	RAD51	ESR1		RPS6KA3
		CDK4	SHMT2	ETS1		SATB1
		CDKN1B	SMAD3	FOS		SLC16A2
		CDKN2A	STMN1	FOXO1		SOX4
		CHEK1	YWHAZ	FOXO3		TMC7
		COPS5		GAS5		YBX3
		CORO1A		GJA1		
		CYP11B2		GNAI2		
		DEDD		GNAI3		
		DHFR		GRB10		
		DHFRP1		ICAM1		
		DND1		KIT		
		DYRK2		MGMT		
		E2F2		MMP1		
		EIF2S3		PLXNC1		
		FAF1		PPP2R2A		
		FBXW7		PRDM1		
		FEN1		PTEN		
		FGF11		RECK		
		FGFR3		RUNX2		
		FSCN1		SELE		
		FURIN		SMAD5		
		GATA3		SOD2		
		H2AFX		SSSCA1		
		HMOX1		SSX2IP		
		HNF4A		STAT5A		
		IFNG		TCEAL1		
		IFNR		TIMP3		
		IL4		TMED7		
		INSIG1		TNFSF10		
		JPH2		TP53		
		LDHA		TRPS1		
		LDHB		VGLL4		
		MAFB				
		MAP3K9				
		MAPK14				
		MAPK7				
		MEN1				
		MLEC				

MMP14
MT1M
MXI1
MYC
NCAN
NCSTN
NDST1
NOS3
NOTCH1
PAK4
PCNA
PDGFRB
POLD1
PRDX6
PRKCH
PSAP
PTPN9
PTPRF
REG4
S100A8
SH3PXD2A
SLC6A4
SP1
SSSCA1
ST7L
STX16
TACC3
TGFB1
TMED7
TMEM92
TNK2
TP53
TRIB3
TRIM11
WNT4
XIAP
ZNF217

hsa-miR-484	hsa-miR-320a-3p	hsa-miR-197-3p	hsa-miR-19b-3p	hsa-miR-214-3p	hsa-miR-99a-5p	hsa-miR-145-5p
4.14	3.47	2.18	1.35	1.32	1.25	1.25
FIS1	ABCG2	BMF	ARID4B	ALPK2	AGO2	ABCC1
SMAD2	AQP1	CD82	ATXN1	ARL2	AKT1	ABHD17C
ZEB1	AQP4	FOXJ2	BACE1	ASF1B	CAPNS1	ABRACL
	AR	FOXO3	BCL2L11	ATF4	CTDSPL	ACTB
	ARF1	MAPK1	BCL3	BAX	FGFR3	ADAM17
	ARPP19	MTHFD1	BMPR2	BCL2L11	FKBP5	ADD3
	BANP	NSUN5	CUL5	BCL2L2	HOXA1	AKR1B10
	BMI1	PMAIP1	CYP19A1	BIRC5	IGF1R	ALDH3A1
	CDK6	RAN	DNMT1	CADM1	MTMR3	ALPPL2
	CRKL	TUSC2	ESR1	CCL5	MTOR	ANGPT2
	CTNNB1		GCM1	CD274	NOX4	AP1G1
	ESRRG		HIPK1	CDK6	RAVER2	APH1A
	FAS		HIPK3	CPD	SERPINE1	ARF6
	FH		KAT2B	CTNNB1	SMARCA5	ARL6IP5
	FOXM1		MTUS1	EZH2	TRIB2	BNIP3
	GNAI1		MXD1	FGFR1		BRAF
	HMGB1		MYCN	GALNT7		C11orf65
	HOXA10		MYLIP	GSR		CAMK1D
	HSPB6		NCOA3	HDGF		CBFB
	IGF1R		PITX1	ING4		CCDC43
	ITGB3		PKNOX1	JAG1		CD28
	KITLG		PPP2R5E	LTF		CD40
	MAPK1		PRKAA1	LZTS1		CD44
	MCL1		PTEN	MAP2K3		CDH2
	MTDH		SMAD4	MAPK1		CDK4
	MYC		SOCS1	MAPK8		CDK6
	NFATC3		TGFBR2	MEF2C		CDKN1A
	NOD2		TLR2	NRAS		CEP19
	NPR1		TNFAIP3	PAPPA		CFTR
	NRP1		TP53	PIM1		CLINT1
	PBX3			PLXNB1		COL5A1
	PDCD4			POR		CPEB4
	PICSAR			POU4F2		CRNDE
	POLR3D			PSMD10		CTGF
	PTEN			PTEN		CTNND1
	RAB11A			QKI		DDC
	RAB14			RAB15		DDX17
	RAC1			SEMA4D		DDX6
	RUNX2			SRGAP1		DFFA
	SUZ12			SRGAP2		DTD1
	TAC1			SUFU		E2F3
	TFRC			TP53		EGFR
	TRPC5			TWIST1		EIF4E
	USP14			UBE2I		EPAS1
	VDAC1			XBP1		ERG
	VEGFA					ESR1
	VIM					ETS1
	YWHAZ					F11R
						FAM3C
						FAM45A
						FLI1

FSCN1
FXN
FZD7
GMFB
GOLM1
HDAC11
HDAC2
HLTF
HMGA2
HOXA9
IFNB1
IGF1R
ILK
IRS1
IRS2
ITGB8
JADE1
JAG1
KLF4
KLF5
KREMEN1
LYPLA2
MAP2K6
MCM2
MDM2
MEST
MIXL1
MMP1
MMP12
MMP14
MSH3
MTDH
MTMR14
MUC1
MYC
MYO5A
MYO6
MYOCD
MYRF
NAIP
NANOG
NDRG2
NDUFA4
NEDD9
NFATC1
NIPSNAP1
NRAS
NUDT1
PAK4
PARP8
PIGF
PODXL
POU5F1
PPM1D
PPP3CA
PTP4A2

PXN
ROBO2
ROCK1
RPA1
RPS6KB1
RREB1
RTKN
SENP1
SERINC5
SERPINE1
SET
SMAD2
SMAD3
SOCS7
SOX2
SOX9
SP1
SP7
SPTBN1
SPTLC1
SRGAP1
STAT1
SWAP70
TGFB2
TGFBR2
TIRAP
TMEM9B
TMOD3
TNFSF13
TPM3
TPRG1
TSPAN6
TUG1
VEGFA
VPS51
YES1

hsa-miR-125b-5p	hsa-miR-627-5p	hsa-miR-342-3p	hsa-miR-409-3p	hsa-miR-21-5p	hsa-miR-106a-5p	hsa-miR-16-5p
1.23	0.99	0.90	0.89	0.88	0.83	0.82
ABTB1	KDM3A	BIRC6	AKT1	ABCB1	APC	ACVR2A
AHRR		BMP7	ANG	AKT2	APP	ADORA2A
AKT1		CTBP2	CTNND1	ANKRD46	ARID4B	AKT3
ALOX5		DNMT1	ELF2	ANP32A	ATG7	APP
ANGPT2		E2F1	FGA	APAF1	ATM	ARHGDIA
APC		GEMIN4	FGB	BASP1	BCL10	ARL2
APLN		ID4	FGG	BCL10	BMP2	AXIN2
ARID3A		IKBKG	FRAT1	BCL2	CASP7	BACE1
ARID3B		MTDH	GAB1	BCL6	CCND1	BCL2
BAK1		SREBF1	IFNG	BMI1	CDKN1A	BDNF
BBC3		SREBF2	MET	BMPR2	CDX2	BIRC5
BCL2		TAB2	MGMT	BTG2	CXCL8	BMI1
BCL2L2		TAB3	NLK	CADM1	CYP19A1	BRCA1
BCL3			PHF10	CASC2	E2F1	CADM1
BMF			RDX	CASP8	ERCC1	CAPRIN1
BMPR1B			RECK	CBX4	FAS	CCND1
BTG2			RSU1	CCL20	FASTK	CCND2
CBFB			STAG2	CCR1	HIF1A	CCND3
CCNJ			UGT2B17	CDC25A	HIPK3	CCNE1
CD44			ZEB1	CDK2AP1	HMGA2	CCNT2
CDH5				CEBPB	IL10	CDK6
CDKN2A				CLU	LIMK1	CDS2
CDKN2D				COL4A1	MAPK9	CHEK1
CEBPA				COX2	MFN2	CHUK
CGN				DAXX	MGST2	CLDN2
CSNK2A1				DDAH1	MYB	FGF2
CYP24A1				DERL1	MYLIP	FGFR1
DGAT1				DNM1L	PTEN	GLS2
DKK3				DOCK4	RARB	HDGF
DRAM2				DOCK5	RB1	HGF
DUSP6				DOCK7	RBL2	HMGA1
E2F2				DUSP10	RND3	HMGA2
E2F3				E2F1	RUNX1	IFNG
EGFR				EGFR	RUNX3	IGF1R
EIF4EBP1				EGLN1	SIRPA	IL12B
EIF5A2				EIF4A2	SLC2A3	KDR
ENPEP				ERBB2	STAT3	KRAS
EPO				FASLG	TGFBR2	MAP7
EPOR				FBXO11	TIMP2	METTL13
ERBB2				FMOD	ULK1	MTOR
ERBB3				FOXO1	VEGFA	MYB
ETS1				FZD6		NCOR2
Fas				GAS5		NCSTN
FES				GDF5		OPRM1
FGFR2				HMGB1		PIM1
FZD6				HNRNPK		PPM1D
GAB2				HPGD		PRDM4
GLI1				ICAM1		PTGS2
GRIN2A				ICOSLG		PURA
GSS				IGF1R		RAF1
HK2				IL12A		RECK

HMGA1	IL1B	RICTOR
HMGA2	IRAK1	RPS6KB1
HOTTIP	ISCU	SLC6A4
ICAM2	JAG1	SOCS3
IGF1R	JMY	SOX5
IGF2	LRP6	SOX6
IKZF2	LRRFIP1	TP53
IKZF3	MAP2K3	TPPP3
IKZF4	MARCKS	UCA1
IL6R	MEF2C	UNG
IRF4	MSH2	VEGFA
JAK2	MSH6	WEE1
KLC2	MSLN	WNT3A
KLF13	MTAP	WNT4
LACTB	MYD88	YAP1
LIFR	NAV3	ZYX
LIN28A	NCAPG	
LIN28B	NCOA3	
LIPA	NFIA	
MAN1B1	NFIB	
MAP3K11	NTF3	
MAPK14	OXTR	
MCL1	PCBP1	
MEGF9	PCGF2	
MMP13	PDCD4	
MMP2	PIAS3	
MMP26	PIK3R1	
MUC1	PLAT	
MXD1	PLOD3	
NCOR2	PPARA	
NES	PPIF	
NEU1	PSMD9	
NKIRAS2	PTEN	
NTRK3	PTPN14	
PCTP	PTX3	
PHF8	RASA1	
PIAS3	RASGRP1	
PIGF	RECK	
PIK3CB	REST	
PIK3CD	RHO	
PODXL	RHOB	
PPP1CA	RPS7	
PRDM1	RTN4	
PRKRA	SATB1	
PTH1R	SERPINB5	
RAF1	SERPINI1	
RPS6KA1	SETD2	
SCNN1A	SIRT2	
SEMA4C	SMAD7	
SET	SMARCA4	
SFRP5	SMN1	
SGPL1	SOCS1	
SIRT7	SOCS6	
SMAD4	SOD3	
SMO	SOX2	
SPHK1	SOX5	

STARD13	SP1
STAT3	SPRY2
SUV39H1	STAT3
TBC1D1	STUB1
TET2	TAP1
TNF	TCF21
TNFAIP3	TGFB2
TP53	TGFBI
TP53INP1	TGFBR2
VDR	TGFBR3
VPS4B	TGIF1
VPS51	TIAM1
	TICAM2
	TIMP3
	TLR3
	TM9SF3
	TNFAIP3
	TNFRSF10B
	TOPORS
	TP53BP2
	TP63
	TPM1
	TRAF7
	UBE2N
	VEGFA
	VHL
	WWP1
	YOD1

hsa-miR-17-5p	hsa-let-7b-5p	hsa-miR-29a-3p	hsa-miR-30c-5p	hsa-miR-221-3p	hsa-miR-92a-3p	hsa-miR-30b-5p
0.82	0.71	0.64	0.61	0.57	0.48	0.44
ABCA1	ACTG1	ABL1	BCL9	ADAM1A	ARID4B	ATG12
ADAR	ACVR1	ADAM12	BECN1	ADAMTS6	BCL2L11	BCL2
APP	AGO1	ADAMTS9	CAMK2D	ANXA1	BMPR2	BCL6
BCL2	AKT2	AHR	CASP3	APAF1	CCL8	BCL9
BCL2L11	ANAPC1	AKT2	CCND2	ARF4	CD69	BECN1
BMP2	CCNA1	AKT3	CDC42	ARIH2	CDH1	CAT
BMPR2	CCNA2	ALDH5A1	CTGF	ARNT	CPEB2	CCNE2
BRCA2	CCND1	ATG9A	DDIT4	ASZ1	DNMT1	CTHRC1
CCL1	CCND2	BACE1	DLL4	BBC3	DUSP10	DLL4
CCND1	CDC25A	BCL2	DNMT1	BCL2L11	ESR2	DNMT1
CCND2	CDC34	BCL7A	EIF2S1	BECN1	FBXW7	EIF2S1
CDKN1A	CDK6	CACNA1C	FASN	BMF	HDAC2	EIF5A2
CLOCK	COL3A1	CALCR	FOXO3	BNIP3	HIPK1	ERG
CLU	CPEB1	CCND1	HDAC4	BNIP3L	HIPK3	HOXA1
CYP7B1	CPEB3	CCND2	HSPA4	BRAP	IKZF1	MBNL1
DAPK3	CPEB4	CCNT2	IDH1	CDKN1B	ITGA5	MBNL2
DNAJC27	CTHRC1	CD276	IER2	CDKN1C	KAT2B	MBNL3
DNMT1	CYP2J2	CD93	IL11	CERS2	KLF2	NOTCH1
E2F1	E2F2	CDC42	JAK1	CORO1A	KLF4	PDGFRB
E2F3	EZH2	CDC7	MCL1	CREBZF	LASP1	RASAL2
EGR2	HMGA1	CDK2	MTA1	CTCF	MAP2K4	RUNX2
EPAS1	HMGA2	CDK4	MTTP	CXCL12	MAPK8	SERPINE1
ETV1	HRAS	CDK6	NCOR2	DDIT4	MAPRE1	SIX1
FBXO31	IFNB1	CEACAM6	NOTCH1	DICER1	MYCBP2	SMAD1
GPR137B	IGF1R	CLDN1	PAK1	DIRAS3	MYLIP	SNAI1
HBP1	IGF2BP1	COL10A1	RARB	DKK2	NR1H4	SOCS1
HIF1A	IGF2BP2	COL1A2	RASAL2	DVL2	OSBPL2	TP53
HSPB2	IRS2	COL3A1	RFX6	ESR1	OSBPL8	
ICAM1	LGR4	COL4A1	RUNX2	ETS1	PCGF5	
IGFBP3	LIN28A	COL4A2	SERPINE1	FMR1	PHLPP1	
ITGB8	LIN28B	COL5A2	SMAD1	FOS	PTEN	
JAK1	LRIG1	CPEB3	SNAI1	FOXO3	RAD21	
KAT2B	MTPN	CPEB4	SNAI2	GJA1	RFFL	
LDLR	NR2E1	CYP2C19	SOCS3	GRB10	RGS5	
LIMK1	NRAS	DICER1	TGIF2	HECTD2	SIRT1	
MAP3K12	PDGFRA	DKK1	TP53	HMGXB4	SOCS5	
MAPK9	PRDM1	DNMT1	TWF1	HOXB5	STAT3	
MDM2	RDH10	DNMT3A	UBE2I	ICAM1	TGFBR2	
MEF2D	RPIA	DNMT3B	VIM	KIT	TP63	
MFN2	TGFBR1	ELN		MBD2		
MMP2	TLR4	FBN1		MDM2		
MYC	TNFRSF10B	FGA		MEOX2		
NABP1		FGB		MGMT		
NCOA3		FGG		MMP2		
NPAS3		FOXO3		MYBL1		
NPAT		FSTL1		NAIP		
PDLIM7		GLUL		PAK1		
PHLPP1		GPR85		PIK3R1		
PKD2		GSK3B		PTEN		
PKNOX1		HBP1		RAB1A		
PPP2R2A		HMGCR		RAD51		

PTEN
PTPRO
RAD21
RB1
RBL1
RBL2
RND3
RUNX1
SELE
SIRPA
SMAD4
SMURF1
SOCS6
STAT3
TBC1D2
TCEAL1
TCF3
TGFBR2
TIMP3
TLR7
TNF
TNFSF12
TP53COR1
TP53INP1
TRIM8
UBE2C
VEGFA
VLDLR
WEE1
YES1
ZBTB4
ZFYVE9
ZNFX1

IFNAR1
IGF1
IMPDH1
ITGA11
ITGA6
ITGB1
ITIH5
KDM5B
KEAP1
KLF4
KREMEN2
LAMC2
LOX
LPL
MCL1
MMP2
MUC1
MYC
MYCN
NASP
NAV3
NFIA
NMI
PDGFRB
PER1
PIK3R1
PPM1D
PPP1R13B
PTEN
PXDN
QKI
RAN
RASGRP1
RNASEL
ROBO1
S100B
SAPCD2
SERPINB9
SERPINH1
SETDB1
SFRP2
SLC22A7
SPARC
SRGAP2
TDG
TET1
TET2
TET3
TFEB
TNFAIP3
TRAF4
TRIM68
VDAC1
VEGFA
ZFP36

RB1
RECK
RUNX1
SELE
SIRT1
SOCS1
SOCS3
SSX2IP
STAT5A
STMN1
TBK1
TCEAL1
TICAM1
TIMP3
TMED7
TNFSF10
TP53
TRPS1
USP18
WEE1
ZEB2

hsa-miR-20a-5p	hsa-miR-132-3p	hsa-miR-618	hsa-miR-138-5p	hsa-miR-382-5p	hsa-miR-663b	hsa-miR-483-5
0.43	0.40	0.35	0.34	0.28	0.27	0.26
ABL2	AGO2		ADGRA2	DRD1	EEF1A2	ALCAM
ANKH	ARHGAP32		AKT1	MXD1	IGF2	CKB
APP	BDNF		ARHGEF3	NFIA		FAM160B2
ARHGAP12	CCNA2		BAG1	PTEN		MAPK3
ATG16L1	CCNB1		BCL11A	YBX1		NOTCH3
BAMBI	CDKN1A		BLCAP			RHOA
BCL2	CRK		CASP3			SRF
BCL2L11	EGFR		CCND1			
BMPR2	FOXO1		CCND3			
BNIP2	GDF5		CD274			
CCND1	HBEGF		CDH1			
CCND2	IRAK4		CYTOR			
CDKN1A	JPT1		EED			
CRIM1	KLHL11		EID1			
DAPK3	MAPK1		EIF4EBP1			
DNMT1	MMP9		EZH2			
DUSP2	MUC13		FERMT2			
E2F1	PIK3R3		FOSL1			
E2F3	RAF1		FOXC1			
EGLN3	RASA1		GNAI2			
EGR2	RB1		H2AFX			
EPAS1	SIRT1		HIF1A			
ETV1	SLC2A1		IGF1R			
FBXO31	SMAD2		KDM5C			
GJA1	SOX4		LCN2			
HIF1A	SOX5		MAP3K11			
IRF2	SOX6		MXD1			
ITGB8	SPRED1		NFKB1			
KIF26B	SPRY1		PTK2			
KIT	TJAP1		RARA			
LIMK1	TLN2		RELN			
MAP2K3	YY1AP1		RHOC			
MAP3K12			RMND5A			
MAP3K5			ROCK2			
MAPK9			S100A1			
MCL1			SENP1			
MEF2D			SIRT1			
MYC			SLC45A3			
NFKBIB			SNAI2			
NRAS			SOX4			
PHLPP2			SOX9			
PKD1			SUZ12			
PKNOX1			TERT			
PPARG			TWIST2			
PPP2R2A			VIM			
PRKG1			YAP1			
PTEN			ZEB2			
PTPRO						
PURA						
RB1						
RB1CC1						

RBL1
RBL2
REST
RGS5
RUNX1
RUNX3
SIRPA
SMAD4
SMAD7
STAT3
TCEAL1
TGFBR1
TGFBR2
TIMP2
TP53INP1
TSG101
UBE2C
VEGFA
WEE1
ZFYVE9

hsa-miR-199a-3p	hsa-miR-520e-3p	hsa-miR-31-5p	hsa-miR-28-3p	hsa-miR-146a-5p	hsa-miR-193a-5p	hsa-miR-34a-5p
0.23	0.21	0.21	0.19	0.19	0.18	0.17
AKT1	CD46	ABCB9	STAT5B	BCLAF1	ERBB2	ACSL1
APOE	EGFR	ARID1A	TP53	BRCA1	IGF2BP1	ACSL4
CAV2	MAP3K14	ARPC5		BRCA2	ING5	AGTR1
CD44	MAP4K4	BAP1		CARD10	MTOR	AIP
CDK7	PFKP	C1QTNF9		CASP7	NLN	AKT1
DNAJA4	ZBTB7A	CASR		CCL5	PIK3R3	ANK3
FLT1		CDK1		CCNA2	SRR	AR
FOXA2		CREG1		CCND1	TFAP2A	ARHGDIB
FUT4		CXCL12		CCND2	TP73	ATG4A
HGF		DACT3		CD40LG	WT1	ATG4B
IGF1		DKK1		CD80		ATG4C
ITGA3		DMD		CDKN1A		ATG4D
KDR		DOCK1		CDKN3		ATG5
MAPK1		E2F2		CFH		ATG7
MAPK14		EMSY		CNOT6L		ATP5S
MAPK8		ETS1		COPS8		AXIN2
MAPK9		FOXO3		COX2		AXL
MET		FOXP3		CPM		BAX
MTOR		FZD3		CXCL12		BCL2
PAK4		GNA13		CXCL8		BECN1
PTGS2		HIF1AN		CXCR4		BIRC5
SMARCA2		HOXC13		DUSP1		BMP7
STK11		ICAM1		EGFR		CACNB3
TFAM		IL25		ELAVL1		CCL22
VEGFA		ITGA5		ERBB4		CCND1
YAP1		JAZF1		FADD		CCND3
ZHX1		KLF13		FAF1		CCNE2
		LATS2		FANCM		CD24
		MAP4K4		FAS		CD44
		MCM2		HOXD10		CDC25A
		MET		ICAM1		CDK4
		MLH1		IL6		CDK6
		MMP16		IRAK1		CDKN2A
		MPRIP		IRAK2		CDKN2C
		NFAT5		IS2		CEBPB
		NUMB		KIF22		Crtc1
		PPP2R2A		L1CAM		CSF1R
		PRKCE		LAMC2		CYBB
		RAB27A		LFNG		DGUOK
		RASA1		LRP2		DLL1
		RDX		MIF		E2F1
		RET		MTA2		E2F3
		RHOA		NFAT5		EPHA5
		RHOBTB1		NFKB1		ERBB2
		SATB2		NOS1		FKBP1B
		SELE		NOTCH1		FLOT2
		SGPP2		NOTCH2		FOS
		SLC1A2		NUMB		FOSL1
		SMAD4		PA2G4		FOXP1
		SOX4		PLAUR		FUT8
		SP7		PRKCE		GALNT7

SPRED1	PTGES2	GAS1
SPRED2	PTGS2	GDF5
SPRY1	RAC1	GFRA3
SPRY3	RARB	GRM7
SPRY4	RHO	HDAC1
SRC	RHOA	HMGB1
STK40	RNF11	HNF4A
STMN1	ROCK1	HNF4G
TBXA2R	SIKE1	HOTAIR
TIAM1	SLPI	IFNB1
WASF3	SMAD2	IGF2BP3
XRCC5	SMAD4	IL6R
YY1	SMN1	IMPA1
	SOS1	IMPDH2
	SOX2	INHBB
	STAT1	JAG1
	TGFB1	KCNH1
	TLR2	KCNH2
	TLR4	KDM4A
	TRAF6	KIT
	UHRF1	KLB
	WASF2	KLF12
		KLF4
		L1CAM
		LDHA
		LEF1
		MAGEA12
		MAGEA2
		MAGEA3
		MAGEA6
		MAP2K1
		MAP3K9
		MDM4
		MET
		MTA2
		MYB
		MYC
		MYCN
		NAMPT
		NANOG
		NLRC5
		NOTCH1
		NOTCH2
		NR4A2
		NUMB
		PAM
		PCBP2
		PDGFRA
		PDGFRB
		PEA15
		PIK3CG
		POU5F1
		PPARA
		PPP1CC
		PPP1R10
		PRKD1

RAD51
RBP2
RCAN1
RICTOR
SIRT1
SIRT6
SIRT7
SMAD4
SNAI1
SOX2
SPI1
SRC
STMN1
STX1A
SYT1
TCF7
TGIF2
TP53
TREM2
ULBP2
VAMP2
VEGFA
WNT1
YY1
ZAP70

hsa-miR-376a-3p	hsa-miR-186-5p
0.17	0.15
ACVR1C	ABCB1
AGO2	AKAP12
ATG4C	CSNK2A1
CASP8	FGF2
CDK2	FOXO1
IGF1R	GJA1
KLF15	HIF1A
MEPE	MAP3K2
PIK3R1	NCSTN
SLC16A1	P2RX7
SRSF11	PAK5
TTK	PPM1B
	PTTG1
	PVT1
	RELA
	SETD2
	TWIST1
	VEGFA
	XIAP
