# Supplementary material for: Molecular Characterization of Secreted Factors and Extracellular Vesicles-Embedded miRNAs from Bone Marrow-Derived Mesenchymal Stromal Cells in Presence of Synovial Fluid from Osteoarthritis Patients
Source: Biology (Basel). 2022 Nov 8;11(11):1632. doi: 10.3390/biology11111632 (PMC9687557; doi:10.3390/biology11111632)
Supplement: Supplementary file 1 [file biology-11-01632-s001.zip › Supplementary Table S6.pdf]

Supplementary Table S6 - Univocal miRNA-mRNA targets

ABCA1
ABCB1
ABCB9
ABCC1
ABCG2
ABHD17C
ABL1
ABL2
ABRACL
ABTB1
ACSL1
ACSL4
ACTB
ACTG1
ACVR1
ACVR1B
ACVR1C
ACVR2A
ADAM12
ADAM17
ADAM1A
ADAMTS6
ADAMTS9
ADAR
ADD3
ADGRA2
ADORA2A
AGO1
AGO2
AGPAT2
AGTR1
AHR
AHRR
AIP
AKAP12
AKR1B10
AKR1C2
AKT1
AKT2
AKT3
ALCAM
ALDH3A1
ALDH5A1
ALOX5
ALPK2
ALPPL2
ANAPC1

ANG
ANGPT2
ANK3
ANKH
ANKRD46
ANP32A
ANXA1
AP1G1
APAF1
APC
APH1A
APLN
APOE
APP
AQP1
AQP4
AR
ARF1
ARF4
ARF6
ARHGAP12
ARHGAP19
ARHGAP32
ARHGDIA
ARHGDIB
ARHGEF3
ARID1A
ARID3A
ARID3B
ARID4B
ARIH2
ARL2
ARL6IP5
ARNT
ARPC5
ARPP19
ASF1B
ASZ1
ATF4
ATG12
ATG16L1
ATG4A
ATG4B
ATG4C
ATG4D
ATG5
ATG7
ATG9A

ATM
ATP5S
ATXN1
AURKB
AXIN2
AXL
BACE1
BAG1
BAK1
BAMBI
BANP
BAP1
BASP1
BAX
BBC3
BCAR1
BCL10
BCL11A
BCL2
BCL2L11
BCL2L2
BCL3
BCL6
BCL7A
BCL9
BCLAF1
BDNF
BECN1
BIRC5
BIRC6
BLCAP
BMF
BMI1
BMP2
BMP7
BMPR1B
BMPR2
BNIP2
BNIP3
BNIP3L
BRAF
BRAP
BRCA1
BRCA2
BTG2
C11orf65
C1QTNF9
CACNA1C

CACNB3
CADM1
CALCR
CAMK1D
CAMK2D
CAPNS1
CAPRIN1
CARD10
CASC2
CASP3
CASP7
CASP8
CASR
CAT
CAV2
CBFB
CBX4
CCDC43
CCL1
CCL20
CCL22
CCL5
CCL8
CCNA1
CCNA2
CCNB1
CCND1
CCND2
CCND3
CCNE1
CCNE2
CCNJ
CCNT2
CCR1
CD24
CD274
CD276
CD28
CD40
CD40LG
CD44
CD46
CD69
CD80
CD82
CD93
CDC25A
CDC34

CDC42
CDC7
CDH1
CDH2
CDH5
CDK1
CDK2
CDK2AP1
CDK4
CDK6
CDK7
CDK9
CDKN1A
CDKN1B
CDKN1C
CDKN2A
CDKN2C
CDKN2D
CDKN3
CDS2
CDX2
CEACAM6
CEBPA
CEBPB
CEP19
CERS2
CFH
CFTR
CGN
CHEK1
CHUK
CKB
CLDN1
CLDN2
CLINT1
CLOCK
CLTC
CLU
CNOT6L
COL10A1
COL1A2
COL3A1
COL4A1
COL4A2
COL5A1
COL5A2
COPS5
COPS8

CORO1A
COX2
CPD
CPEB1
CPEB2
CPEB3
CPEB4
CPM
CREBZF
CREG1
CRIM1
CRK
CRKL
CRNDE
Crtc1
CSF1R
CSNK2A1
CTBP2
CTCF
CTDSP2
CTDSPL
CTGF
CTHRC1
CTNNB1
CTNND1
CUL2
CUL5
CXCL12
CXCL8
CXCR4
CYBB
CYP11B2
CYP19A1
CYP24A1
CYP2C19
CYP2J2
CYP7B1
CYTOR
DACT3
DAPK3
DAXX
DDAH1
DDC
DDIT4
DDX17
DDX6
DEDD
DERL1

DFFA
DGAT1
DGUOK
DHFR
DHFRP1
DICER1
DIRAS3
DKK1
DKK2
DKK3
DLL1
DLL4
DMD
DNAJA4
DNAJC27
DND1
DNM1L
DNMT1
DNMT3A
DNMT3B
DOCK1
DOCK4
DOCK5
DOCK7
DRAM2
DRD1
DTD1
DUSP1
DUSP10
DUSP2
DUSP6
DVL2
DYRK2
E2F1
E2F2
E2F3
EED
EEF1A2
EGFR
EGLN1
EGLN3
EGR1
EGR2
EID1
EIF2S1
EIF2S3
EIF4A2
EIF4E

EIF4EBP1
EIF5A2
ELAVL1
ELF2
ELN
EMSY
ENPEP
EP300
EPAS1
EPHA5
EPO
EPOR
ERBB2
ERBB3
ERBB4
ERCC1
ERG
ESR1
ESR2
ESRRG
ETS1
ETV1
EZH2
F11R
FADD
FAF1
FAM160B2
FAM3C
FAM45A
FANCM
FAS
FASLG
FASN
FASTK
FBN1
FBXO11
FBXO31
FBXW7
FEN1
FERMT2
FES
FGA
FGB
FGF11
FGF2
FGFR1
FGFR2
FGFR3

FGG
FH
FIS1
FKBP1B
FKBP5
FLI1
FLOT2
FLT1
FMOD
FMR1
FOS
FOSL1
FOXA2
FOXC1
FOXJ2
FOXM1
FOXO1
FOXO3
FOXP1
FOXP3
FRAT1
FSCN1
FSTL1
FURIN
FUT4
FUT8
FXN
FZD3
FZD6
FZD7
GAB1
GAB2
GALNT7
GAS1
GAS5
GATA3
GCM1
GDF5
GEMIN4
GFRA3
GJA1
GLI1
GLS2
GLUL
GMFB
GNA13
GNAI1
GNAI2

GNAI3
GOLM1
GPR137B
GPR85
GRB10
GRIN2A
GRM7
GSK3B
GSR
GSS
H2AFX
HBEGF
HBP1
HDAC1
HDAC11
HDAC2
HDAC4
HDGF
HECTD2
HGF
HIF1A
HIF1AN
HIPK1
HIPK3
HK2
HLTF
HMGA1
HMGA2
HMGB1
HMGCR
HMGXB4
HMOX1
HNF4A
HNF4G
HNRNPK
HOTAIR
HOTTIP
HOXA1
HOXA10
HOXA9
HOXB5
HOXC13
HOXD10
HPGD
HRAS
HSPA4
HSPB2
HSPB6

ICAM1
ICAM2
ICOSLG
ID4
IDH1
IER2
IFNAR1
IFNB1
IFNG
IFNR
IGF1
IGF1R
IGF2
IGF2BP1
IGF2BP2
IGF2BP3
IGFBP3
IKBKG
IKZF1
IKZF2
IKZF3
IKZF4
IL10
IL11
IL12A
IL12B
IL1A
IL1B
IL25
IL4
IL6
IL6R
ILK
IMPA1
IMPDH1
IMPDH2
ING4
ING5
INHBB
INSIG1
IRAK1
IRAK2
IRAK4
IRF2
IRF4
IRS1
IRS2
IS2

ISCU
ITGA11
ITGA3
ITGA5
ITGA6
ITGB1
ITGB3
ITGB8
ITIH5
JADE1
JAG1
JAK1
JAK2
JAZF1
JMY
JPH2
JPT1
KAT2B
KCNH1
KCNH2
KDM3A
KDM4A
KDM5B
KDM5C
KDR
KEAP1
KIF22
KIF26B
KIT
KITLG
KLB
KLC2
KLF12
KLF13
KLF15
KLF2
KLF4
KLF5
KLHL11
KRAS
KREMEN1
KREMEN2
L1CAM
LACTB
LAMC2
LASP1
LATS2
LCN2

LDHA
LDHB
LDLR
LEF1
LFNG
LGR4
LIFR
LIMK1
LIN28A
LIN28B
LIPA
LOX
LPL
LRIG1
LRP2
LRP6
LRRC8A
LRRFIP1
LTF
LYPLA2
LZTS1
MAFB
MAGEA12
MAGEA2
MAGEA3
MAGEA6
MAN1B1
MAP2K1
MAP2K3
MAP2K4
MAP2K6
MAP3K11
MAP3K12
MAP3K14
MAP3K2
MAP3K5
MAP3K9
MAP4K4
MAP7
MAPK1
MAPK14
MAPK3
MAPK7
MAPK8
MAPK9
MAPRE1
MARCKS
MAX

MBD2
MBNL1
MBNL2
MBNL3
MCL1
MCM2
MDM2
MDM4
MEF2C
MEF2D
MEGF9
MEN1
MEOX2
MEPE
MEST
MET
METTL13
MFN2
MGMT
MGST2
MIF
MIXL1
MLEC
MLH1
MMP1
MMP12
MMP13
MMP14
MMP16
MMP2
MMP26
MMP9
MPRIP
MSH2
MSH3
MSH6
MSLN
MT1M
MTA1
MTA2
MTAP
MTDH
MTHFD1
MTMR14
MTMR3
MTOR
MTPN
MTTP

MTUS1
MUC1
MUC13
MXD1
MXI1
MYB
MYBL1
MYC
MYCBP2
MYCN
MYD88
MYLIP
MYO5A
MYO6
MYOCD
MYRF
NABP1
NAIP
NAMPT
NANOG
NASP
NAV3
NCAN
NCAPG
NCOA3
NCOR2
NCSTN
NDRG2
NDST1
NDUFA4
NEDD9
NES
NEU1
NF1
NFAT5
NFATC1
NFATC3
NFIA
NFIB
NFKB1
NFKBIB
NIPSNAP1
NKIRAS2
NLK
NLN
NLRC5
NMI
NOD2

NOS1
NOS3
NOTCH1
NOTCH2
NOTCH3
NOX4
NPAS3
NPAT
NPR1
NR1H4
NR2E1
NR4A2
NRAS
NRP1
NSUN5
NTF3
NTRK3
NUDT1
NUMB
OPRM1
OSBPL2
OSBPL8
OXTR
P2RX7
PA2G4
PAK1
PAK4
PAK5
PAM
PAPPA
PARP8
PBX3
PCBP1
PCBP2
PCGF2
PCGF5
PCNA
PCTP
PDCD4
PDGFRA
PDGFRB
PDLIM7
PEA15
PER1
PFKP
PHF10
PHF8
PHLPP1

PHLPP2
PIAS3
PICSAR
PIGF
PIK3CB
PIK3CD
PIK3CG
PIK3R1
PIK3R3
PIM1
PITX1
PKD1
PKD2
PKNOX1
PLAT
PLAU
PLAUR
PLOD3
PLXNB1
PLXNC1
PMAIP1
PODXL
POLD1
POLR3D
POR
POU4F2
POU5F1
PPARA
PPARG
PPIF
PPM1B
PPM1D
PPP1CA
PPP1CC
PPP1R10
PPP1R13B
PPP2R2A
PPP2R5E
PPP3CA
PRAP1
PRDM1
PRDM4
PRDX6
PRKAA1
PRKCE
PRKCH
PRKD1
PRKG1

PRKRA
PSAP
PSMD10
PSMD9
PTEN
PTGES2
PTGS2
PTH1R
PTK2
PTP4A2
PTPN14
PTPN9
PTPRF
PTPRO
PTTG1
PTX3
PURA
PVT1
PXDN
PXN
QKI
RAB11A
RAB14
RAB15
RAB1A
RAB27A
RAC1
RAD21
RAD51
RAF1
RAN
RARA
RARB
RASA1
RASAL2
RASGRP1
RAVER2
RB1
RB1CC1
RBL1
RBL2
RBP2
RCAN1
RDH10
RDX
RECK
REG4
RELA

RELN
REST
RET
RFFL
RFX6
RGS5
RHO
RHOA
RHOB
RHOBTB1
RHOC
RICTOR
RMND5A
RNASEL
RND3
RNF11
ROBO1
ROBO2
ROCK1
ROCK2
RPA1
RPIA
RPS6KA1
RPS6KA3
RPS6KB1
RPS7
RREB1
RSU1
RTKN
RTN4
RUNX1
RUNX2
RUNX3
RXRA
S100A1
S100A8
S100B
SAPCD2
SATB1
SATB2
SCNN1A
SELE
SEMA4C
SEMA4D
SENP1
SERINC5
SERPINB5
SERPINB9

SERPINE1
SERPINH1
SERPINI1
SET
SETD2
SETDB1
SFRP2
SFRP5
SGPL1
SGPP2
SH3PXD2A
SHMT2
SIKE1
SIRPA
SIRT1
SIRT2
SIRT6
SIRT7
SIX1
SLC16A1
SLC16A2
SLC1A2
SLC22A7
SLC2A1
SLC2A3
SLC45A3
SLC6A4
SLPI
SMAD1
SMAD2
SMAD3
SMAD4
SMAD5
SMAD7
SMARCA2
SMARCA4
SMARCA5
SMN1
SMO
SMURF1
SNAI1
SNAI2
SOCS1
SOCS3
SOCS5
SOCS6
SOCS7
SOD2

SOD3
SOS1
SOX2
SOX4
SOX5
SOX6
SOX9
SP1
SP7
SPARC
SPHK1
SPI1
SPRED1
SPRED2
SPRY1
SPRY2
SPRY3
SPRY4
SPTBN1
SPTLC1
SRC
SREBF1
SREBF2
SRF
SRGAP1
SRGAP2
SRR
SRSF11
SSSCA1
SSX2IP
ST7L
STAG2
STARD13
STAT1
STAT3
STAT5A
STAT5B
STK11
STK40
STMN1
STUB1
STX16
STX1A
SUFU
SUV39H1
SUZ12
SWAP70
SYT1

TAB2
TAB3
TAC1
TACC3
TAP1
TBC1D1
TBC1D2
TBK1
TBXA2R
TCEAL1
TCF21
TCF3
TCF7
TDG
TERT
TET1
TET2
TET3
TFAM
TFAP2A
TFEB
TFRC
TGFB1
TGFB2
TGFBI
TGFBR1
TGFBR2
TGFBR3
TGIF1
TGIF2
TIAM1
TICAM1
TICAM2
TIMP2
TIMP3
TIRAP
TJAP1
TLN2
TLR2
TLR3
TLR4
TLR7
TM9SF3
TMC7
TMED7
TMEM92
TMEM9B
TMOD3

TNF
TNFAIP3
TNFRSF10B
TNFSF10
TNFSF12
TNFSF13
TNK2
TOPORS
TP53
TP53BP2
TP53COR1
TP53INP1
TP63
TP73
TPM1
TPM3
TPPP3
TPRG1
TRAF4
TRAF6
TRAF7
TREM2
TRIB2
TRIB3
TRIM11
TRIM68
TRIM8
TRPC5
TRPS1
TSG101
TSPAN6
TTK
TUG1
TUSC2
TWF1
TWIST1
TWIST2
UBE2C
UBE2I
UBE2N
UCA1
UGT2B17
UHRF1
ULBP2
ULK1
UNG
USP14
USP18

VAMP2
VDAC1
VDR
VEGFA
VGLL4
VHL
VIM
VLDLR
VPS4B
VPS51
WASF2
WASF3
WEE1
WNT1
WNT3A
WNT4
WT1
WWP1
XBP1
XIAP
XRCC5
YAP1
YBX1
YBX3
YES1
YOD1
YWHAZ
YY1
YY1AP1
ZAP70
ZBTB4
ZBTB7A
ZEB1
ZEB2
ZFP36
ZFYVE9
ZHX1
ZNF217
ZNFX1
ZYX
